# Effects of prime-boost strategies on the protective efficacy and immunogenicity of a PLGA (85:15)-encapsulated *Chlamydia* recombinant MOMP nanovaccine

**DOI:** 10.1093/femspd/ftae004

**Published:** 2024-06-11

**Authors:** Rajnish Sahu, Richa Verma, Timothy E Egbo, Guillermo H Giambartolomei, Shree R Singh, Vida A Dennis

**Affiliations:** Center for NanoBiotechnology Research, Department of Biological Sciences, 1627 Harris Way, Alabama State University, Montgomery AL, 36104, United States; Center for NanoBiotechnology Research, Department of Biological Sciences, 1627 Harris Way, Alabama State University, Montgomery AL, 36104, United States; US Army Medical Research Institute of Infectious Diseases, Unit 8900, DPO, AE, Box 330, 09831, United States; Instituto de Inmunología, Genética y Metabolismo (INIGEM). CONICET. AV. Cordoba 2351, Universidad de Buenos Aires, Buenos Aires, C1120AAR, Argentina; Center for NanoBiotechnology Research, Department of Biological Sciences, 1627 Harris Way, Alabama State University, Montgomery AL, 36104, United States; Center for NanoBiotechnology Research, Department of Biological Sciences, 1627 Harris Way, Alabama State University, Montgomery AL, 36104, United States

**Keywords:** PLGA [poly (d, l-lactic-co-glycolide)] nanoparticles, immunization routes, IFN-γ, *Chlamydia*

## Abstract

To begin to optimize the immunization routes for our reported PLGA-rMOMP nanovaccine [PLGA-encapsulated *Chlamydia muridarum* (Cm) recombinant major outer membrane protein (rMOMP)], we compared two prime-boost immunization strategies [subcutaneous (SC) and intramuscular (IM-p) prime routes followed by two SC-boosts)] to evaluate the nanovaccine-induced protective efficacy and immunogenicity in female BALB/c mice. Our results showed that mice immunized via the SC and IM-p routes were protected against a Cm genital challenge by a reduction in bacterial burden and with fewer bacteria in the SC mice. Protection of mice correlated with rMOMP-specific Th1 (IL-2 and IFN-γ) and not Th2 (IL-4, IL-9, and IL-13) cytokines, and CD4^+^ memory (CD44^high^CD62L^high^) T-cells, especially in the SC mice. We also observed higher levels of IL-1α, IL-6, IL-17, CCL-2, and G-CSF in SC-immunized mice. Notably, an increase of cytokines/chemokines was seen after the challenge in the SC, IM-p, and control mice (rMOMP and PBS), suggesting a Cm stimulation. In parallel, rMOMP-specific Th1 (IgG2a and IgG2b) and Th2 (IgG1) serum, mucosal, serum avidity, and neutralizing antibodies were more elevated in SC than in IM-p mice. Overall, the homologous SC prime-boost immunization of mice induced enhanced cellular and antibody responses with better protection against a genital challenge compared to the heterologous IM-p.

## Teaser

*Chlamydia* nanovaccine development and optimization of immunization routes.

## Introduction

Development of a safe and effective vaccine is only the first step to control a human infectious pathogen. In recent years, vaccine development efforts against human pathogens are rapidly progressing toward biodegradable nanoparticle-based delivery of encapsulated subunit antigens (Lung et al. 2020). Due to the weak immunogenicity of subunit vaccines, they require a delivery system, an adjuvant to bolster immune responses, and often multiple dosages to induce adequate protective immune responses (Singh et al. 2015, Tsoras and Champion 2019, Kumar et al. 2020). Vaccine-induced immunity is also profound upon the immunization route to elicit anamnestic immune responses (Estcourt et al. 2005, Herzog 2014, Pais et al. 2019). Targeting mucosal routes for immunization has been effective since most infections occur through mucosal surfaces (Holmgren and Czerkinsky 2005). Nevertheless, multiple studies have indicated that nonmucosal routes can induce better immune responses for vaccines against some cancers (Chen et al. 2018, 2019), bacteria (Khan et al. 2017, Khademi et al. 2019), viruses (Lin et al. 2014, Gebauer et al. 2019), and parasites (Noormehr et al. 2018, Pandey et al. 2018). Currently, many subunit vaccines that are in preclinical development interchangeably use systemic and mucosal routes or a combination of both for immunization (Holmgren et al. 2013, 2018, Manoff et al. 2015, Kumar et al. 2020, Lakatos et al. 2020).

*Chlamydia trachomatis* (Ct) is the most common etiologic agent of bacterial sexually transmitted diseases, leading to considerable reproductive morbidities worldwide (Low et al. 2016). Generally, females are more prone to long-term persistent infections that pose significant risks, often causing pelvic inflammatory disease, infertility, ectopic pregnancy, and chronic abdominal pain (Cluver et al. 2017, Poston et al. 2019). Reportedly, *Chlamydia* infections can boost human immunodeficiency virus (HIV) transmission and may serve as a cofactor in human papillomavirus-induced cervical neoplasia (Simonetti et al. 2009, Jensen et al. 2014, Masia et al. 2020), thereby posing a considerable burden on public health globally. Despite these significant public health challenges, there is no approved chlamydial vaccine. The development of a vaccine against genital *Chlamydia* could greatly aid in the amelioration of the induced morbidities and comorbidities.

*Chlamydia* is an infectious pathogen whereby vaccine-induced immunity is exceptionally challenging since an attenuated or inactivated whole chlamydial elementary bodies (EBs) vaccine is not practical due to the induction of immunopathology (Mabey et al. 2014). Also, the need for serovar-specific protection (de la Maza et al. 2017) further stifles the process. Available evidence indicates that protection against *Chlamydia* involves coordination from cell-mediated and humoral immunity such as CD4^+^ T-cells, Th1-secreting cytokines (i.e. IFN-γ, IL-2), and antibodies (IgG and IgA), to clear the bacterial infection (Farris et al. 2010, Fiorino et al. 2013, Lorenzen et al. 2015, Wern et al. 2017). Other investigators show that an immunomodulatory Th17 response also plays a role in *Chlamydia* vaccine-induced immunity (Vicetti Miguel et al. 2016).

*Chlamydia* major outer membrane protein (MOMP) has been studied for years and is a prime subunit vaccine target because it is immunogenic and elicits cellular and humoral immune responses that are requisites for protective immunity against genital *Chlamydia* (O’Meara et al. 2013, de la Maza et al. 2017, Poston et al. 2019). Recombinant MOMP adjuvanted with DDA/MPL and chlamydial Pmps (Yu et al. 2014), CAF01, and CAF09 (Pal et al. 2017), TLR agonists (Cheng et al. 2011, 2014, Pal et al. 2020, Tifrea et al. 2020) or cholera toxin subunits (Singh et al. 2006, Ekong et al. 2009) have all protected mice against genital *Chlamydia*. Nonetheless, the protection afforded by the MOMP vaccine candidates is short-term and does not induce sterilizing or long-lasting protective immunity, probably because of ineffective adjuvants to bolster mucosal immune responses (Singh et al. 2006, Stary et al. 2015), efficient delivery systems (Dixit et al. 2018), or inadequate routes of administration (Fiorino et al. 2013, Lorenzen et al. 2015, Pais et al. 2019).

The vaccine-delivery route has a significant impact on the induction of efficacious host immune responses. Moreover, an optimal vaccine-delivery system can profoundly dictate the outcome of the elicited immune responses. Efforts to develop and optimize a vaccine against *Chlamydia* have sought numerous delivery routes (Berry et al. 2004, Ralli-Jain et al. 2010, Pais et al. 2017) and prime-boost immunization strategies (Brown et al. 2012, Lorenzen et al. 2015, Badamchi-Zadeh et al. 2016). Our effort in the preclinical development of a *Chlamydia* vaccine has focused primarily on delivery systems using biodegradable-polymeric nanoparticles with self-adjuvanting properties. Using such an adjuvant-delivery system, we have successfully developed several potential *Chlamydia* nanovaccine candidates against MOMP or its peptides (Taha et al. 2012, Fairley et al. 2013, Dixit et al. 2014, Verma et al. 2018, Sahu et al. 2020). We recently reported that our chlamydial PLGA-rMOMP nanovaccine consisting of recombinant major outer membrane protein (rMOMP) encapsulated in extended-releasing PLGA (85:15) nanoparticles-triggered activation of dendritic cells to produce robust Th1 cytokines, adaptive immune responses, and MHC-II antigen presentation (Sahu et al. 2020). Our data showed that PLGA-rMOMP administered via a homologous prime-boost subcutaneous (SC) route protected mice against a *Chlamydia muridarum* (Cm) genital challenge but failed to confer complete protection. Given the impact of prime-boost immunization routes on a vaccine’s protective potential, herein, we determined the impact of the SC homologous versus a heterologous intramuscular (IM-p) prime-boost immunization on the PLGA-rMOMP nanovaccine-induced immunogenicity and protective efficacy against a Cm genital challenge. Here, we present and discuss our results from the prime-boost studies conducted in the female BALB/c mouse model.

## Materials and methods

### Reagents

Cm [strain Nigg II; previously called *C. trachomatis* mouse pneumonitis (MoPn) biovar] expressed as inclusion forming units (IFU/ml) was purchased from Virusys Corporation (Taneytown, MD, USA). The mouse-derived McCoy fibroblasts cell line and Dulbecco’s Modified Eagle’s Medium (DMEM) with high glucose and l-glutamine were both purchased from American Type Culture Collection (ATCC) (Manassas, VA, USA). PLGA polymer (85:15 poly-lactide: poly-glycolide), dichloromethane (DCM), polyvinyl alcohol (PVA), and mitomycin-C were purchased from Sigma-Aldrich (St Louis, MO, USA). ELISA MAX^TM^ Deluxe kits for IL-2 and IFN-γ were purchased from BioLegend^®^ Inc. (San Diego, CA, USA). Anti-CD 90.2 magnetic beads and MACS columns were purchased from Miltenyi Biotech (Auburn, CA, USA). CellTrace^TM^ CFSE (carboxyfluorescein succinimidyl ester) cell proliferation assay kit (C34554), Remel^TM^ PathoDx^TM^  *Chlamydia* culture confirmation kit (R62210), RPMI-1640 with GlutaMax^TM^ and HEPES, heat-inactivated fetal bovine serum (FBS), and ACK lysing solution were purchased from ThermoFisher Scientific (Waltham, MA, USA). The Fc block anti-CD16/32 antibody (BD:553141), fluorochrome-conjugated antibodies: CD3-APC-Cy7 (BD:560590), CD4-PerCP-Cy5.5 (BD:550954), CD62L-APC (BD:553152), CD44-PE (BD:553134), and BD OptEIA kits IL-1α, IL-6, and IL-4 were obtained from BD Biosciences (San Jose, CA, USA). Cytokines/chemokines Bio-plex assays were purchased from Bio-Rad (Hercules, CA, USA). Medroxyprogesterone acetate (Depo-Provera) was purchased from Pfizer (New York, NY, USA). Cycloheximide was obtained from EMD Biosciences (La Jolla, CA, USA).

### PLGA-rMOMP nanovaccine formulation

The PLGA-rMOMP nanovaccine was formulated, as previously reported (Sahu et al. 2020, 2021). Briefly, PLGA 85:15 (150 mg) was dissolved in DCM, followed by the addition of 2 mg of rMOMP, homogenization, and then the addition of 1% PVA. The resulting double-emulsion was gently stirred overnight at room temperature (RT) to allow evaporation of the DCM organic solvent, harvested by ultracentrifugation, washed, and then lyophilized in the presence of a 5% trehalose solution. Lyophilized nanoparticles were stored at −80°C in a sealed container until used.

### Mice immunization and challenge

**Figure 1.**
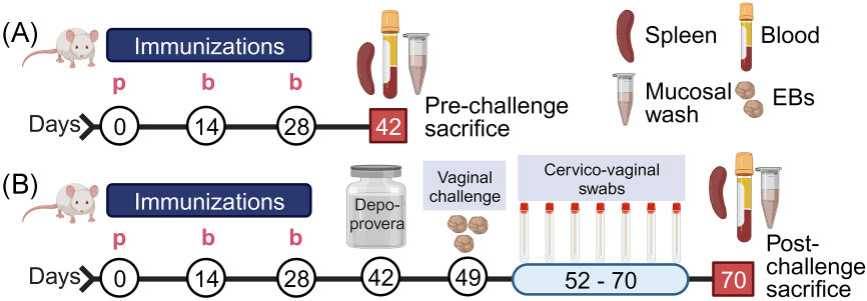
Schematic of immunization and challenge. (A) Female BALB/c mice (six per group) were each given PLGA-rMOMP (50 µg) on day 0 for priming (p) via the IM-p (heterologous) or SC (homologous) routes. IM-p and SC mice each received two boosters (b) immunization of PLGA-rMOMP (50 µg) via the SC route on days 14 and 28 and then sacrificed on day 42 (six per group) for immunogenicity studies. (B) For efficacy studies, immunized mice (six per group) were each challenged via the intravaginal route with live Cm IFU (1 × 10^5^) on day 49, followed by a collection of cervico-vaginal swabs at 3-day intervals up to 3-weeks to quantify Cm IFU followed by sacrifice on day 70. Some mice received rMOMP (50 µg) or PBS via the SC route to serve as controls. After each sacrifice (days 42 and 70), spleens (for T-cells), blood (for serum), and mucosal washes were collected to evaluate cellular and humoral immune responses before and after a challenge. (Illustration created in Biorender.com).

Female BALB/c mice (4–6 weeks old) were purchased from Charles River Laboratory (Raleigh, NC, USA) and housed under standard pathogen-free and controlled environmental conditions and provided with food and water *ad libitum*. Mice were acclimatized for 2-weeks before all experimental procedures as approved by Alabama State University Institutional Animal Care and Use Committee (IACUC). Mice were divided into experimental groups (12 mice/group) for the immunization studies and were primed on day 0 via the IM-p (heterologous) or SC (homologous) routes with PLGA-rMOMP (50 µg). Two boosters of PLGA-rMOMP (50 µg) were administered via the SC route on days 14 and 28 (Fig. 1A). A total of 2-weeks following the last immunization (day 42), 6 mice/group were sacrificed to collect spleen, serum, and mucosal wash samples for analyses of cellular and humoral immune responses, respectively. Mice in the PBS and rMOMP groups, respectively, were administered SC with 100 µl of sterile PBS or 50 µg of rMOMP.

For the challenge studies, immunized mice (six per/group) were each administered 2.5 mg of Depo-Provera (day 42) SC and challenged (day 49) intravaginally with 1 × 10^5^ IFU of Cm in sucrose phosphate glutamate (SPG) buffer (Verma et al. 2018, Sahu et al. 2021). Cervico-vaginal swabs were collected at 3-day intervals for 3-weeks, and mice were sacrificed on day 70 (Fig. 1B). All swabs were collected in SPG buffer and stored at −80°C to quantify the Cm vaginal bacterial burden (Verma et al. 2018, Sahu et al. 2021).

### Quantification of Cm from vaginal swabs

Swabs were propagated in McCoy cell monolayers containing 0.5 µg/ml cycloheximide, centrifuged at 750 × *g* for 1 h at RT and then incubated for 2 h at 37°C in a 5% CO_2_ humidified atmosphere. After that, the media was replaced with fresh media containing 0.5 µg/ml cycloheximide and further incubated for 30 h. The cells were washed, fixed in 95% ethanol, and stained with a FITC-labeled *Chlamydia* antibody using the Remel^TM^ PathoDx^TM^  *Chlamydia* Culture confirmation kit. Inclusions were captured using a fluorescent microscope (Nikon, Melville, NY, USA), visually counted and calculated as IFU/ml (Verma et al. 2018, Sahu et al. 2021).

### Antigen-specific T-cells proliferation, and memory and effector phenotypes quantification

Spleens were pooled per group and kept in RPMI-1640 supplemented with 10% FBS and antibiotics–antimycotic. Single-cell suspensions were obtained and filtered through a 40-micron nylon mesh strainer and washed before red blood cells lyses using ACK lysing solution. Total T-cells were purified from splenocytes with anti-CD 90.2-conjugated magnetic beads by positive selection over MACS columns and subjected to CFSE-based proliferation assay, as previously described (Dixit et al. 2018, Verma et al. 2018, Sahu et al. 2020, 2021). CFSE-labeled T-cells (1 × 10^6^) were co-cultured with mitomycin-C (25 µg/ml) treated APCs (1 × 10^6^) and stimulated with rMOMP (5 µg/ml) in round bottom-polypropylene tissue culture tubes and incubated for 120 h at 37°C in a 5% CO_2_-humidified atmosphere. Cells were harvested and stained using CD3-APC-Cy7, CD4-PerCP-Cy5.5, CD62L-APC, and CD44-PE to evaluate T-cells proliferation, and memory (CD44^high^ CD62L^high^) and effector (CD44^high^ CD62L^low^) phenotypes. Following the staining, cells were washed, fixed, and data were acquired on a BD LSR II flow cytometer and analyzed using FCS Express FLOW6 (De Novo Software, Pasadena, CA, USA). Gating on CFSE^+^ T-cells was used for the selection of CD3^+^CD4^+^ T-cell populations (Figures S1–S8, Supporting Information). Histogram fluorescence intensities were used to quantify the proliferating and resting T-cells amongst the total CFSE^+^CD3^+^CD4^+^ T-cells.

### Cytokines quantification

Purified T-cells were co-cultured with APCs and stimulated with rMOMP (5 μg/ml), and cell-free culture supernatants were collected at 120 h by centrifugation for cytokines quantification, as described previously (Dixit et al. 2018, Verma et al. 2018, Sahu et al. 2020, 2021). The Th1 and Th2 cytokine ratios were calculated using the following equation:


\begin{eqnarray*}
\textit{Ratio} = \frac{{Th1{\rm}\left( {IL - 2{\mathrm{\,\,}}or{\mathrm{\,\,}}IFN - \gamma } \right)}}{{Th2{\rm}\left( {IL - 4} \right)}}.
\end{eqnarray*}

### Quantification of antigen-specific serum and mucosal antibody isotypes

Antibody isotypes (IgG2a and IgG2b (Th1) and IgG1 (Th2)) A were quantified from pooled sera or vaginal wash (including IgA) samples, as described previously (Singh et al. 2006, Fairley et al. 2013, Dixit et al. 2014, Verma et al. 2018, Sahu et al. 2021). Briefly, ELISA plates were coated with 100 µl (1 µg/ml) of purified rMOMP and kept overnight at 4°C. The rMOMP-coated plates were then washed with PBS-Tween 20 (PBST) and blocked in 3% nonfat dry milk. In a separate plate, samples were serially diluted (2-fold), starting at 1:4000 (serum IgG1), 1:500 (serum IgG2a and IgG2b), 1:25 (mucosal wash IgG1, IgG2a, and IgG2b), and 1:5 (mucosal wash IgA) to determine the endpoint titers. Antigen-specific IgG2a and IgG2b (Th1) and IgG1 (Th2) antibodies were detected using isotype-specific HRP-conjugated antibodies (goat antimouse) and TMB substrate. The endpoint titer was considered to be the last sample dilution with readings higher than the mean + 5 standard deviations (SD) of the negative control serum or vaginal wash (IgG isotypes) or the mean + 3 (SD) of the negative control vaginal wash samples (IgA). All samples were run in triplicates, and experiments were repeated at least three times. The Th1 and Th2 antibody ratios were calculated using the following equation:


\begin{eqnarray*}
\textit{Ratio} = \frac{{Th1\left( {IgG2a\,\,or\,\,IgG2b} \right)}}{{Th2\left( {IgG1} \right)}}.
\end{eqnarray*}

### Quantification of antibody isotypes avidity index

Serum antibody isotypes avidity index (AI) was determined as previously described (Verma et al. 2018, Sahu et al. 2020, 2021). ELISA plates were coated with purified rMOMP, as described above in the serum and mucosal antibodies section. Sera were diluted (1:50, 1:100, 1:200, and 1:400) and then added to wells in parallel (two sets per plate) and incubated for 2 h at RT. Plates were washed with PBST, and one set for each sample was treated with urea (8 M in PBST), and the other set was treated with PBST for 5 min at RT. After washing, rMOMP-specific IgG2a and IgG2b (Th1) and IgG1 (Th2) isotypes AI was detected using isotype-specific HRP-conjugated goat antimouse antibodies and TMB substrate. The experiments were repeated at least two times, and each sample was run in triplicates. The AI was calculated using the following equation:


\begin{eqnarray*}
AI\,\,\left( \% \right) = \left( {\frac{{OD\,\,\textit{with}\,\,\textit{urea}}}{{OD\,\,\textit{without}\,\,\textit{urea}}}} \right)100.
\end{eqnarray*}

### Neutralization of *Chlamydia in vitro*

Neutralization of Cm EBs by sera from immunized (pre) and immunized-challenged (post) mice was performed in McCoy cells, as previously described (Verma et al. 2018, Sahu et al. 2021). Briefly, McCoy cell monolayers were infected with EBs (pretreated with sera) by centrifugation for 1 h at 750 × *g* and incubated for 30 h in a 37°C incubator. Cells were fixed, stained with *Chlamydia* confirmation kit (Remel, ThermoFisher, USA). Inclusions were captured using a fluorescent microscope (Nikon, Melville, NY, USA), counted and calculated as IFU/ml.

### Statistical analysis

Data were analyzed by two-way analysis of variance (ANOVA) followed by Tukey’s multiple comparison to compare the number of Cm IFU, and cellular and humoral immune responses from rMOMP, SC and IM-p using GraphPad Prism 10 (San Diego, CA, USA) to observe the differences between immunized (pre), immunized-challenged (post) and control groups. One-way ANOVA was used for the average of total IFU to compare the % reduction in recovered IFU between immunized-challenged (post) groups. *P*-values ≤ .05 were considered statistically significant.

## Results

### Homologous (SC) is more effective than heterologous (IM-p) prime-boost immunization against clearance of genital *Chlamydia*

**Figure 2.**
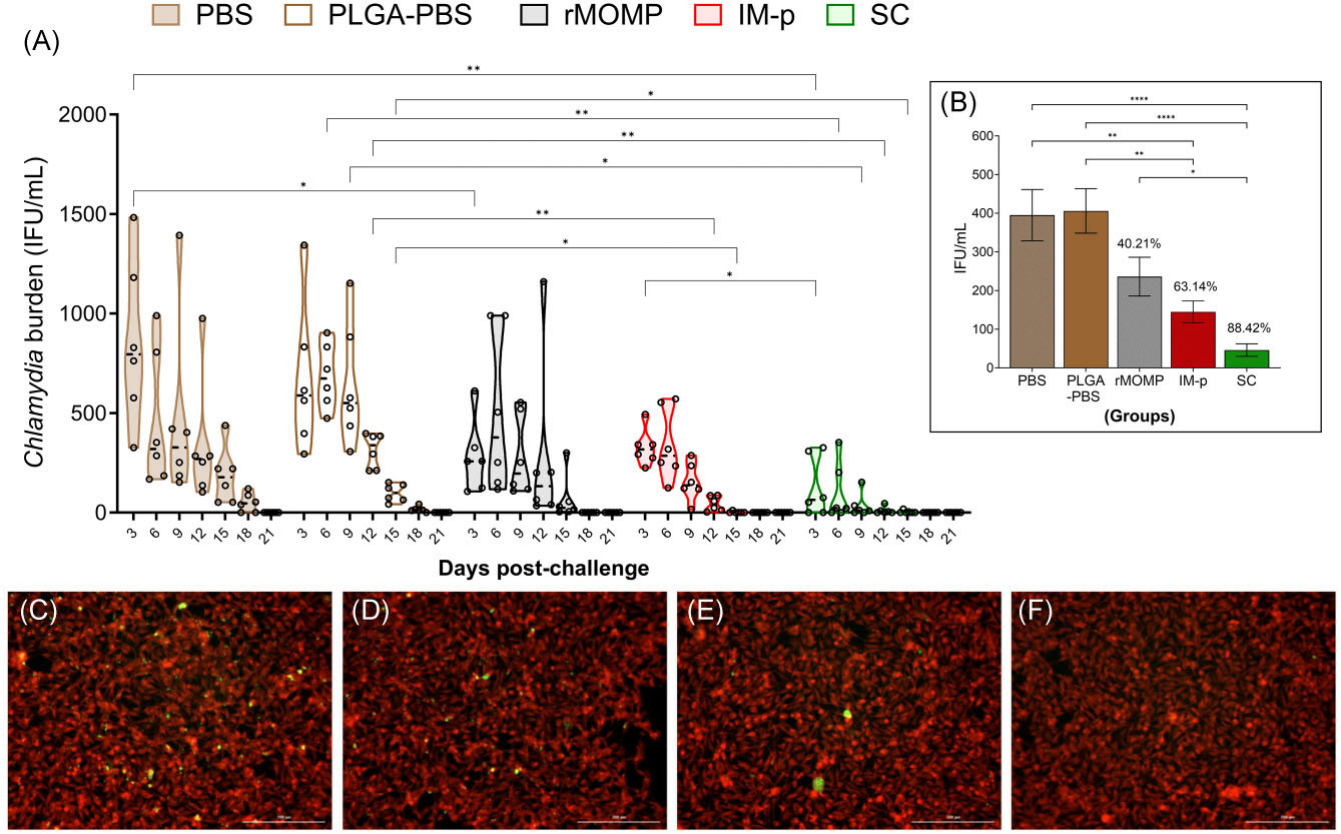
SC rather than the IM-p prime-boost immunization provides better protection against a genital chlamydial challenge. Mice were each given PLGA-rMOMP (50 µg) as priming via the IM-p (heterologous) or SC (homologous) routes. IM-p and SC mice each received two booster immunization of PLGA-rMOMP (50 µg) via the SC route at 2-week intervals and then challenged via the intravaginal route with live Cm IFU (1 × 10^5^). Cervico-vaginal swabs were collected at 3-day intervals up to 3-weeks and propagated in McCoy fibroblasts to quantify recovered Cm IFU from swabs. (A) Each floating bar represents the minimum and maximum range for the IFU counts (IFU/ml) from individual swabs, and the horizontal middle dotted line represents the mean of IFU/ml for each group of mice after challenge. (B) Graph insert represents the average of total IFU/ml (mean ± SE) calculated for each group between days 3 and 18, and presented as a % reduction of IFU compared to the PBS control. Immunofluorescence microscopic visualization of Cm IFU (green) cytoplasm in fibroblasts (red). Fibroblasts were exposed to swabs collected from mice on day 12 after the challenge. (C) PBS, (D) rMOMP, (E) IM-p, and (F) SC groups. Statistical analyses were performed using two-way ANOVA followed by Tukey’s Post-test (A) and average IFU comparison was performed using one-way ANOVA (B). Significant differences in IFU counts were considered at *P < .05, **P < .01, and ****P < .0001. No exclusions were applied for IFU counts.

Exploring various prime-boost immunization strategies in the preclinical development of an efficacious vaccine against genital *Chlamydia* is essential (Brown et al. 2012, Badamchi-Zadeh et al. 2016). To begin to optimize the immunization routes for our chlamydial PLGA-rMOMP nanovaccine, we used two prime-boost immunization strategies to compare the nanovaccine-induced protective efficacy and immunogenicity in mice. As depicted in Fig. 1, mice received PLGA-rMOMP either via the IM-p (heterologous) or SC (homologous) routes followed by two SC route booster immunizations and then a challenge via the mucosal intravaginal route with Cm IFU (1 × 10^5^). Cervico-vaginal swabs were collected at 3-day intervals up to 3-weeks to evaluate protection by quantifying the recovered IFU from swabs. Both SC and IM-p immunizations confer significant protection (*P* < .05–.01) against genital *Chlamydia* in comparison to the rMOMP and PBS control groups, by their lower IFU and faster bacterial clearance (Fig. 2A). Of note, SC mice had significantly less IFU (*P* < .01) with accelerated bacterial clearance compared to the IM-p mice. An average of the total recovered IFU/group (days 3–18) revealed the lowest recovered IFU in the SC group with 88.42% reduction versus IM-p (63.14%) and rMOMP (40.21%) as compared with the PBS control (Fig. 2B). These findings indicate that the homologous SC route affords the best protection of mice against a Cm genital challenge. Visualization of Cm in fibroblasts employing immunofluorescence microscopy validated the lower IFU in the SC, followed by IM-p and then the rMOMP and PBS groups (Fig. 2C–F).

### Nanovaccine-induced antigen-specific cellular immune responses in mice

It is well-known that cell-mediated immunity, as elicited by a vaccine, is key to protecting against genital *Chlamydia* with activated T-cells and Th1 cytokines serving as pivotal protagonists (Bakshi et al. 2018, Helble et al. 2020). We evaluated T-cell-mediated immune effectors that may correlate with PLGA-rMOMP protective efficacy against genital *Chlamydia* in immunized mice. Purified splenic T-cells from immunized (pre) and immunized-challenged (post) mice were co-cultured with mitomycin-C treated APCs and stimulated with rMOMP for 120 h. Post-stimulation, cell-free supernatants were collected and used to quantify various cytokines/chemokines that are necessary for clearance of chlamydial burden in the genital tract (Helble et al. 2020).

**Figure 3.**
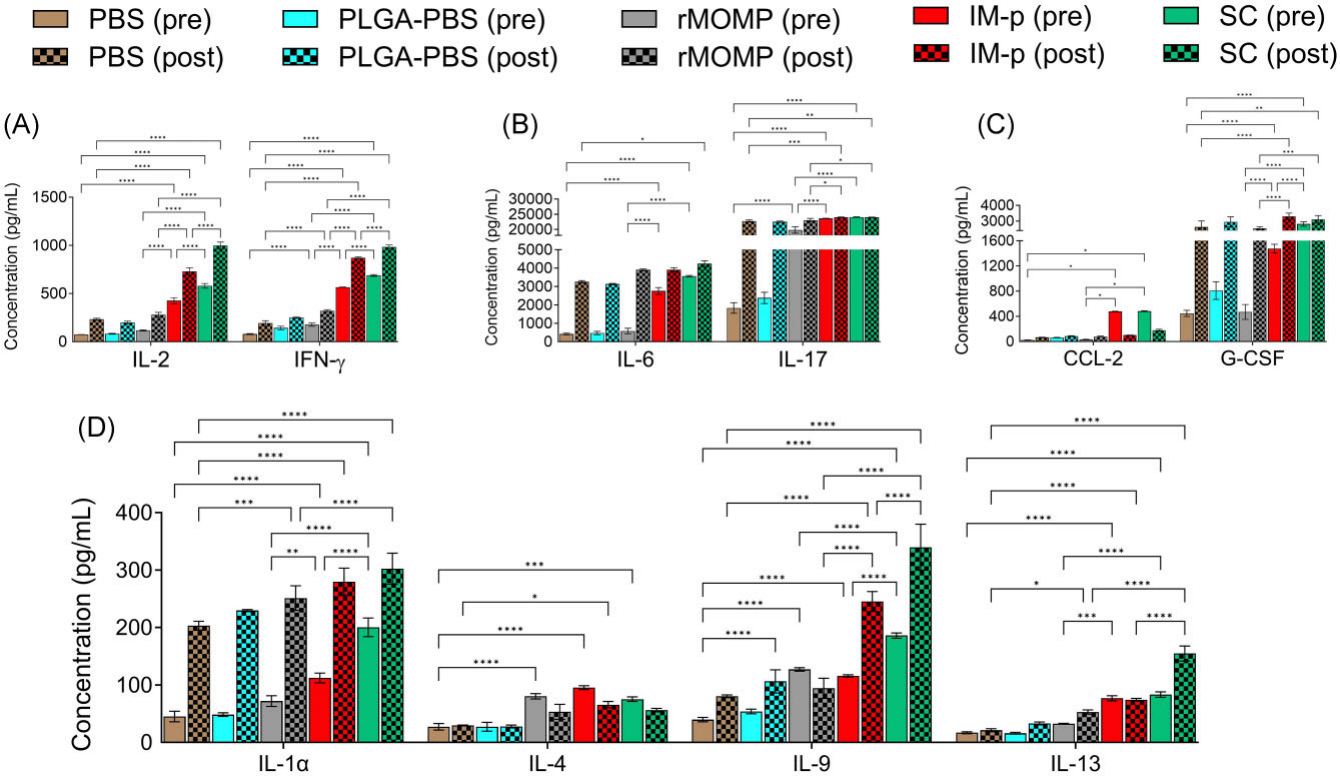
Production of rMOMP-specific cytokines and chemokines after immunization (pre), challenge (post). Mice were immunized and challenged, as shown in Fig. 1. Immunomagnetic purified splenic T-cells (1 × 10^6^) from immunized (pre) and immunized-challenged mice (post) were cocultured with mitomycin-C treated APCs (1 × 10^6^) and stimulated with rMOMP (5 µg/ml) for 120 h at 37°C in a 5% CO_2_-humidified atmosphere. Cell-free supernatants were collected by centrifugation and used for quantification of cytokines. (A) IL-2, IFN-γ (B) IL-6, IL-17, (C) CCL-2, G-CSF, and (D) IL-1α, IL-4, IL-9, and IL-13. Statistical analyses were performed using two-way ANOVA followed by Tukey’s Post-test. Significance was established at *P < .05 **P < .01, ***P < .001, and ****P < .0001.

As depicted in Fig. 3(A), T-cells from SC- and IM-p-immunized mice (pre) were found to significantly (*P* < .0001) produce more Th1 cytokines (IL-2, and IFN-γ) compared to those from the rMOMP and other control groups of mice. It is worth noting that both cytokines were significantly enhanced (*P* < .0001) after a Cm genital challenge (post).

We also observed (Fig. 3B) that T-cells from the SC and IM-p immunized mice (pre) produced significantly (*P* < .001) higher levels of rMOMP-specific IL-6 (a pro-inflammatory cytokine) compared to the control groups (pre). After a genital challenge (post), T-cells from all groups secreted high levels of IL-6, with SC mice showing the highest level. IL-17 production was significantly high (*P* < .0001) in the rMOMP-, SC-, and IM-p-immunized (pre) groups. Lower levels of IL-17 was observed in the negative control groups. We also observed enhanced IL-17 production (> 8-fold) in all groups of mice after the Cm genital challenge (post).

Figure 3(C) shows that CCL-2 (also known as monocyte chemoattractant protein 1 (MCP1)) production was significantly (*P* < .05) induced in SC- and IM-p-immunized mice (pre), which decreased after Cm genital challenge (post). The granulocyte colony-stimulating factor (G-CSF) (Fig. 3C), a stimulator of stem cells to produce more leukocytes, was significantly (*P* < .0001) high in SC- and IM-p-immunized mice (pre). In addition, we noticed that the SC mice produced 2-fold higher levels of G-CSF in comparison to the IM-p mice. The production of G-CSF was significantly (*P* < .0001) enhanced after a Cm genital challenge (post) but interestingly, slightly higher in IM-p mice compared to SC mice (post).

In Fig. 3(D), it can be seen that T-cells from SC- and IM-p-immunized mice (pre) secreted significantly (*P* < .0001) higher IL-1α than the rMOMP and other control groups. After challenge (post), the levels of IL-1α were significantly (*P* < .0001) enhanced for SC, followed by IM-p and rMOMP compared to the negative control groups. The production of IL-4, a prototype Th2 cytokine, was low in SC-, IM-p-, and rMOMP-immunized mice (pre), and was further reduced in challenge mice (post). On the other hand, IL-13 production was elevated significantly (*P* < .001) in SC (post) compared to all other groups (pre and post). Additionally, IL-9 production was significantly (*P* < .0001) induced in all pre and post groups, especially in SC. Overall, Th2 cytokines were lower than Th1 cytokines in SC and IM-p mice. The PLGA-PBS negative control responses were similar to those of PBS; thus, thus statistical comparison was not included in the graphs for clarity.

**Figure 4.**
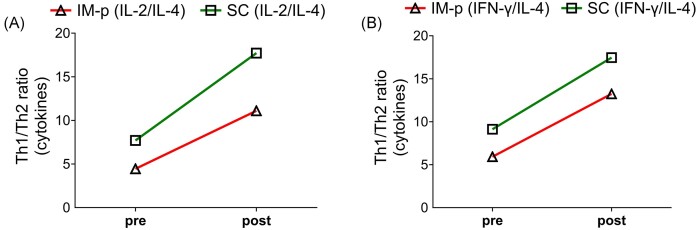
Th1, Th2 cytokines ratio. The Th1 (IL-2 or IFN-γ)/Th2 (IL-4) ratios were calculated between the groups immunized (pre) or immunized-challenged (post) and plotted as comparison between pre and post. Ratio (A) (IL-2/IL-4) and (B) (IFN-γ/IL-4).

Further analyses of the Th1 (IL-2, IFN-γ) and Th2 (IL-4) cytokines ratio (Fig. 4A and B) revealed that the SC immunized mice (pre) produced higher rMOMP-specific Th1 (IL-2/IL-4; 7.70 and IFN-γ/IL-4; 9.13) cytokines compared to the IM-p (IL-2/IL-4; 4.45 and IFN-γ/IL-4; 5.93), which were than enhanced after a Cm genital challenge (post) in SC (IL-2/IL-4: 17.71) and (IFN-γ/IL-4; 17.46) and IM-p (IL-2/IL-4; 11.10 and IFN-γ/IL-4; 13.24). Whereas, the rMOMP (not shown in Fig. 4) immunized mice (pre) produced Th1 (IL-2/IL-4; 1.47 and IFN-γ/IL-4; 2.2) cytokines and slightly enhanced Th1 (IL-2/IL-4; 5.26 and IFN-γ/IL-4; 6) after a Cm genital challenge (post). Overall, these results demonstrate that the SC homologous prime-boost induced higher Th1 cytokine responses to enhance the protection against a genital *Chlamydia* challenge. The PBS control group comparison (pre and post) was not included (Fig. 4) due to low or no significant change in IL-4 production (Fig. 3D) after a Cm challenge.

**Figure 5.**
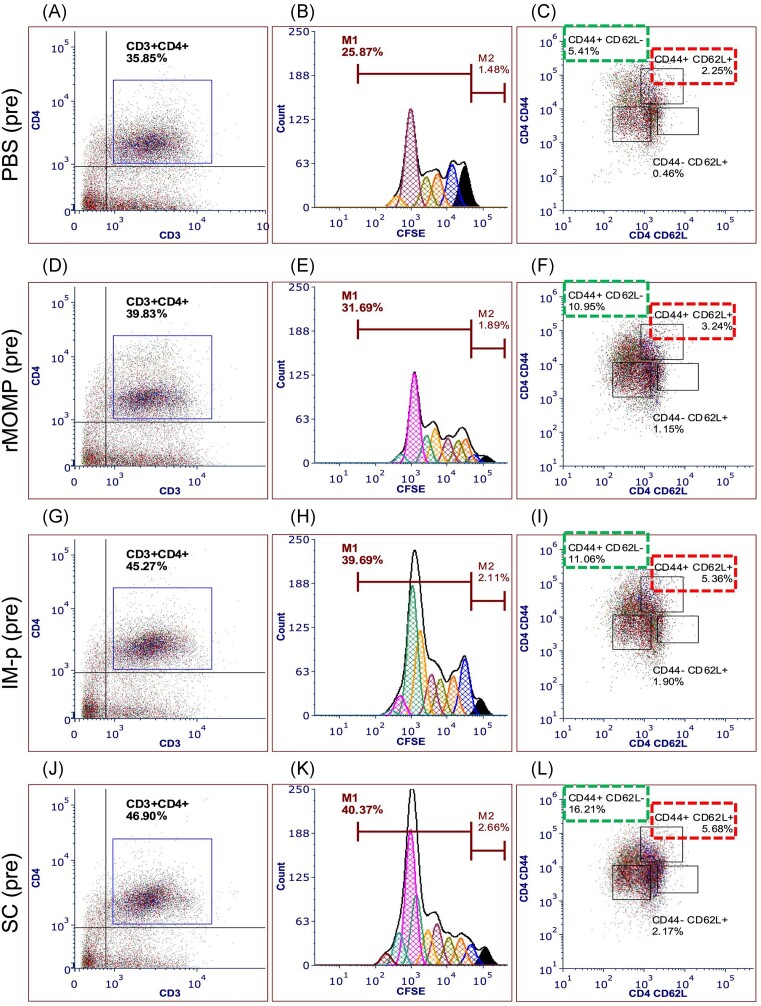

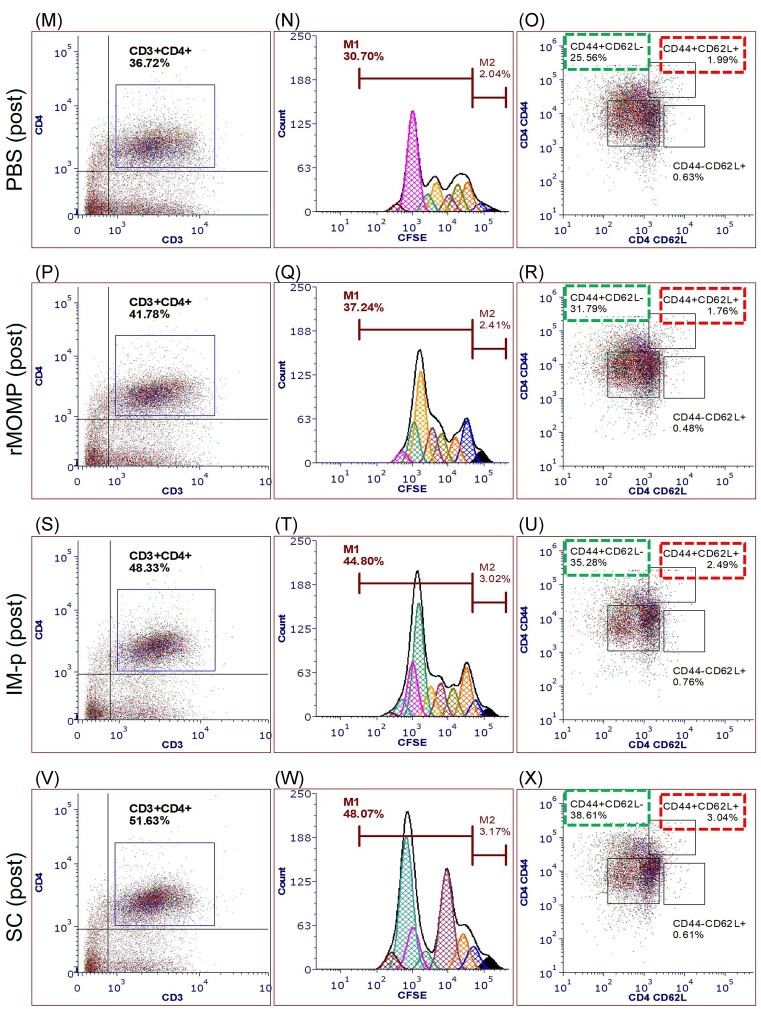
Chlamydia-specific CD4^+^ T-cells proliferation and memory and effector phenotypes in immunized and immunized-challenged mice. Groups of mice were immunized and challenged, as described in Fig. 1 legend. Immunomagnetic purified splenic T-cells (1 × 10^6^) from immunized (pre) and immunized-challenged mice (post) were cocultured with mitomycin-C treated APCs (1 × 10^6^) and stimulated with rMOMP (5 µg/ml) for 120 h at 37°C in a 5% CO_2_-humidified atmosphere. Cocultures were centrifuged, and cells were stained with fluorochrome-labeled specific antibodies for CD3, CD4, CD44, and CD62 L surface markers. Cells were acquired on a flow cytometer and analyzed by gating on CD3^+^ T-cells with secondary gating on CFSE^+^CD3^+^CD4^+^ T-cells for proliferating memory (CD44^+^ CD62L^+^) and effector (CD44^+^ CD62L^−^) T-cells phenotypes. Immunized mice (pre); (A, B, and C) PBS, (D, E, and F) rMOMP, (G, H, and I) IM-p, and (J, K, and L) SC. Immunized-challenged mice (post); (M, N, and O) PBS, (P, Q, and R) rMOMP, (S, T, and U) IM-p, and (V, W, and X) SC groups. (C, F, I, L, O, R, U, and X) Dotted red box; CD4 memory (CD44^+^ CD62L^+^) T-cells % population, Dotted green box; effector (CD44^+^ CD62L^−^) T-cells % population.

Assessment of a chlamydial vaccine efficacy also entails evaluating the vaccine’s capacity to induce activation of T-cells and the formation of memory and effector cells. Here, the ability of PLGA-rMOMP to activate T-cells after immunization and challenge of mice was investigated. We focused on CD4^+^ T-cells proliferation and differentiation into memory (CD44^high^ CD62L^high^), and effector (CD44^high^ CD62L^low^) phenotypes that contribute to bacterial clearance. Our comparative results, as depicted in Fig. 5(A)–(L), show that after immunization (pre), T-cell activation was in the order of magnitude SC > IM-p > rMOMP > PBS. The increase in the CD4^+^ T-cell percentages was comparable for SC (46.90%) and IM-p (45.27%) but higher than those of the rMOMP (39.83%) and PBS (35.85%) groups (Fig. 5A, D, G, and J). Proliferating CD4^+^ T-cells (M1) were also similar and higher in numbers between the SC (40.37%) and IM-p (39.69%) than the rMOMP (31.69%) and PBS (25.87%) mice (Fig. 5B, E, H, and K). An essential, memory (CD44^high^ CD62L^high^) T-cell phenotype was induced by immunization of mice in both the SC (5.68%) and IM-p (5.36%), compared to the rMOMP (3.24%) and PBS (2.25%) groups (Fig. 5C, F, I, and L). However, the CD4^+^ effector (CD44^high^ CD62L^low^) phenotype was higher for the SC (16.21%) compared to IM-p (11.06%), rMOMP (10.95%) and PBS (5.41%) groups (Fig. 5C, F, I, and L).

Similarly, activated CD4^+^ T-cells increased more in the SC and IM-p mice after a genital challenge (Fig. 5M, P, S, and V), followed by the rMOMP and PBS mice. CD4^+^ T-cell numbers increased in the sequential order of magnitude SC > IM-p > rMOMP > PBS (51.63%, 48.33%. 41.78%, and 36.72%), along with heightened proliferation (respectively, 48.07%, 44.80%, 37.24%, and 30.70%) (Fig. 5N, Q, T, and W). The impact of a Cm challenge was also evident with the induction of more effector CD4^+^ T-cells in mice being higher in SC (38.61%), followed by IM-p (35.28%), rMOMP (31.79%), and PBS (25.56%) groups (Fig. 5O, R, U, and X). Conversely, a reduction of the CD4^+^ T-cells memory phenotype was seen after challenge in SC (3.04%), IM-p (2.49%), rMOMP (1.76%), and PBS (1.99%) mice due to the higher numbers of effector cells (Fig. 5O, R, U, and X). Together these findings suggest that both prime-boost strategies elicited cell-mediated immune effectors that correlated with their protected status. However, the SC homologous prime-boost induced the highest cellular immune effectors that possibly enhanced the protection of mice against a genital *Chlamydia* challenge.

### Nanovaccine-induced antigen-specific serum and mucosal antibodies in mice

**Figure 6.**
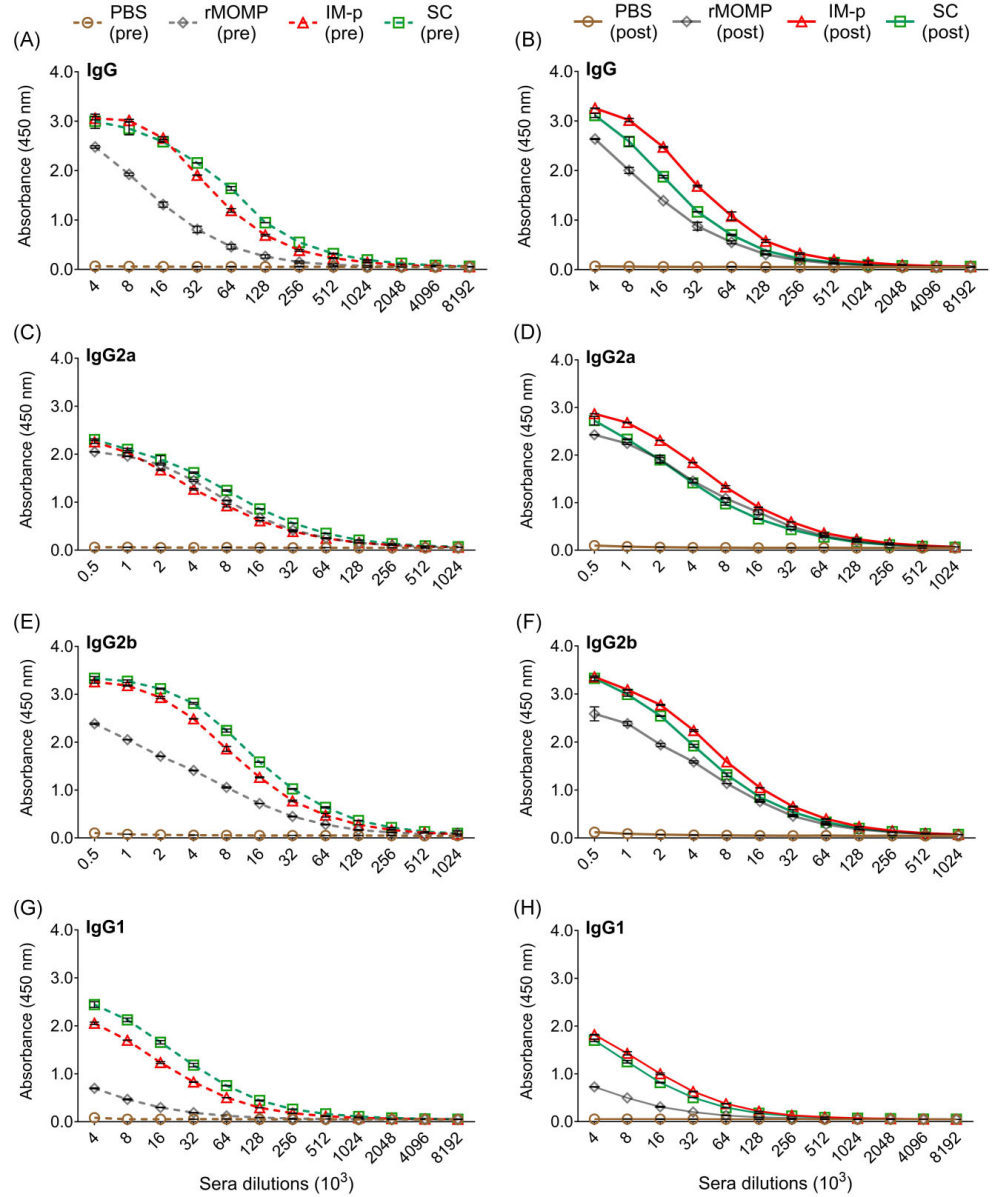
Production of systemic rMOMP-specific antibodies after immunization (pre) and challenge (post). Groups of mice were immunized and challenged, as described in the legend of Fig. 1. Sera collected from groups of immunized (pre), and immunized-challenged (post) mice were pooled per group and used to quantify rMOMP-specific antibody isotypes by ELISA. Immunized mice (pre); (A) IgG, (C) IgG2a, (E) IgG2b, and (G) IgG1, and immunized-challenged mice (post); (B) IgG, (D) IgG2a, (F) IgG2b, and (H) IgG1. Sera were diluted at a 2-fold serial dilution to determine the endpoint antibody isotype titers. Each data point represents the mean ± SD of triplicate samples.

Next, we evaluated the humoral protective immunity induced in mice by measuring antigen-specific total IgG and Th1 (IgG2a and IgG2b) and Th2 (IgG1) systemic and mucosal antibody isotypes before and after a chlamydial genital challenge. Sera obtained from immunized (pre), and immunized-challenged (post) mice were pooled per group to quantify rMOMP-specific antibody isotype endpoint titers by ELISA. Our results show that SC or IM-p mice produced elevated (2-fold or more) IgG antibodies (pre or post) compared to the rMOMP mice (pre or post) (Fig. 6A and B; Table 1). Higher Th1 (IgG2a and IgG2b) and Th2 (IgG1) IgG antibody isotypes were produced in the SC than the IM-p mice after immunization with both exhibiting a mixed Th1/Th2 antibody profile. The rMOMP-immunized mice (pre) also produced predominant Th1 than Th2 antibodies (Fig. 6C, E, and G; Table 1). After a genital challenge (post), all antibody isotypes receded in the SC, but the IM-p mice had an enhanced Th1 with reduced Th2 antibodies. Only IgG2b was increased in rMOMP mice after challenge while other isotypes remained unchanged (Fig. 6D, F, and H; Table 1). Except for a weak IgG2b production after a genital challenge, the PBS control mice (pre) did not produce antigen-specific antibodies (Fig. 6A–H; Table 1).

**Table 1.** Antigen-specific serum antibody endpoint titers of immunized (pre) and immunized-challenged (post) mice.

Antibodies	PBS		rMOMP		IM-p		SC	
Serum	Pre	Post	Pre	Post	Pre	Post	Pre	Post
IgG	–	–	256 000	256 000	1 024 000	1 024 000	1 024 000	512 000
IgG2a	–	–	128 000	128 000	128 000	256 000	256 000	128 000
IgG2b	–	500	128 000	256 000	128 000	256 000	512 000	256 000
IgG1	–	–	32 000	32 000	256 000	128 000	512 000	128 000

**Figure 7.**
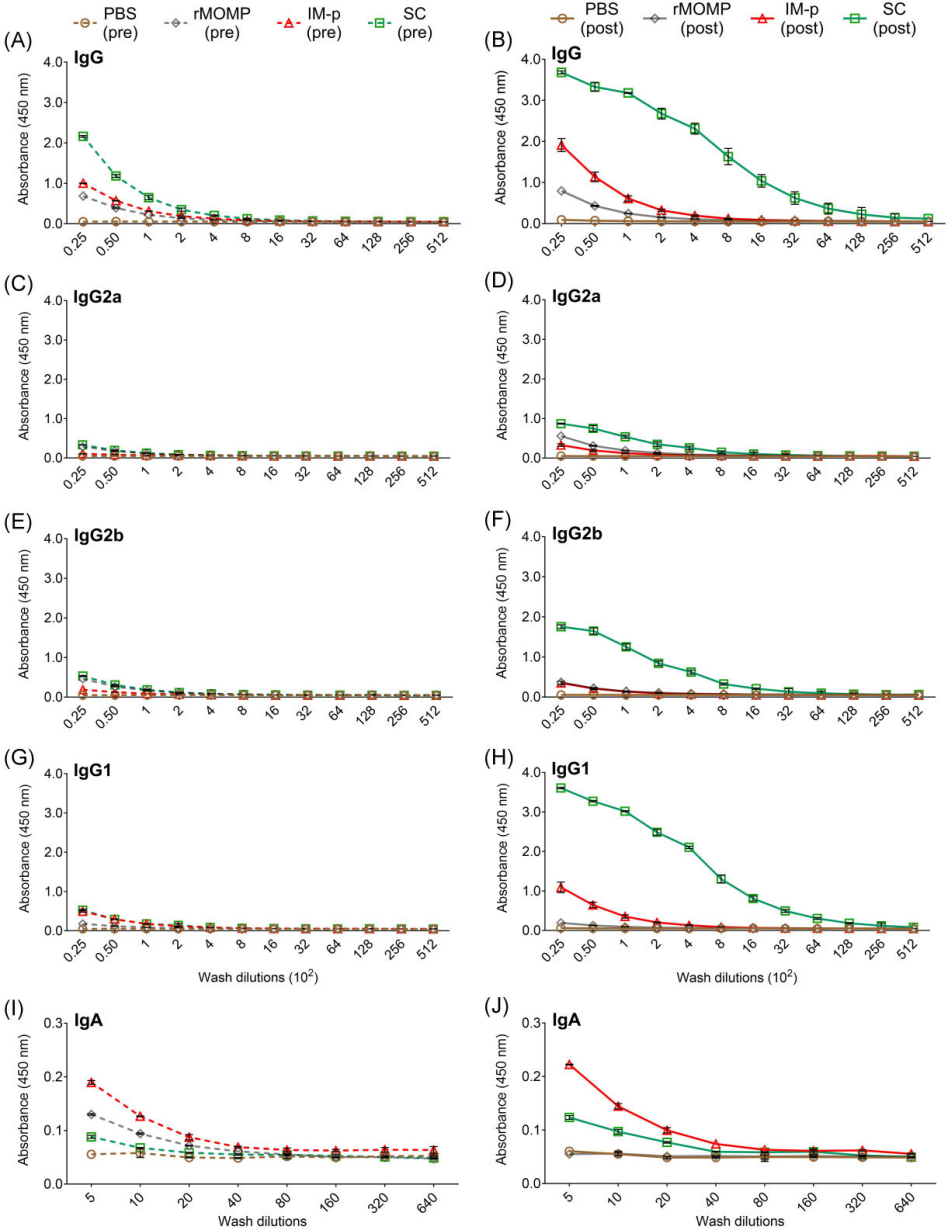
Production of mucosal rMOMP-specific antibodies after immunization (pre) and challenge (post). Groups of mice were immunized and challenged, as described in Fig. 1 legend. Vaginal washes collected from groups of immunized (pre) and immunized-challenged (post) mice were pooled per group and used to quantify rMOMP-specific antibody isotypes by ELISAs. Immunized mice (pre); (A) IgG, (C) IgG2a, (E) IgG2b, (G) IgG1, and (I) IgA, and immunized-challenged mice (post) groups; (B) IgG, (D) IgG2a, (F) IgG2b, (H) IgG1, and (J) IgA. Mucosal washes were diluted at a 2-fold serial dilution to determine the antibody isotype endpoint titers. Each data point represents the mean ± SD of triplicate samples.

Further, we evaluated rMOMP-specific mucosal antibodies by collecting mucosal washes from immunized (pre) and immunized-challenged (post) mice, as described above for sera. We observed (Fig. 7A and B; Table 2) that SC and IM-p immunizations (pre) induced 2–4-fold higher IgG antibody titers that increased by 128-fold after challenge in SC (post) relative to the rMOMP mice. Collectively, all groups of immunized mice (pre) produced low mucosal IgG isotypes (Fig. 7C, E, and G). The data shows an interesting pattern for mucosal responses in SC (post) with marked increase of Th1 (IgG2a; 2-fold and IgG2b; 16-fold), Th2 (IgG1; 128-fold), and IgA (2-fold) antibodies, which were not seen in other groups (Fig. 7D, F, and H; Table 2). PBS mice (pre) did not produce antigen-specific mucosal antibodies (Fig. 7A–J; Table 2).

**Table 2.** Antigen-specific mucosal antibody endpoint titers of immunized (pre) and immunized-challenged (post) mice.

Antibodies	PBS		rMOMP		IM-p		SC	
Serum	Pre	Post	Pre	Post	Pre	Post	Pre	Post
IgG	–	–	100	200	200	400	400	25 600
IgG2a	–	–	50	100	–	50	100	800
IgG2b	–	–	100	100	50	50	100	1600
IgG1	–	–	25	25	100	200	100	12 800
IgA	–	–	10	–	20	20	5	10

Overall, our results demonstrate that the PLGA delivery system enhanced the production of Th1 and Th2 antibodies against rMOMP at both systemic and mucosal sites in mice. More importantly, the SC homologous prime-boost strategy was more effective in eliciting robust humoral protective immunity.

**Figure 8.**
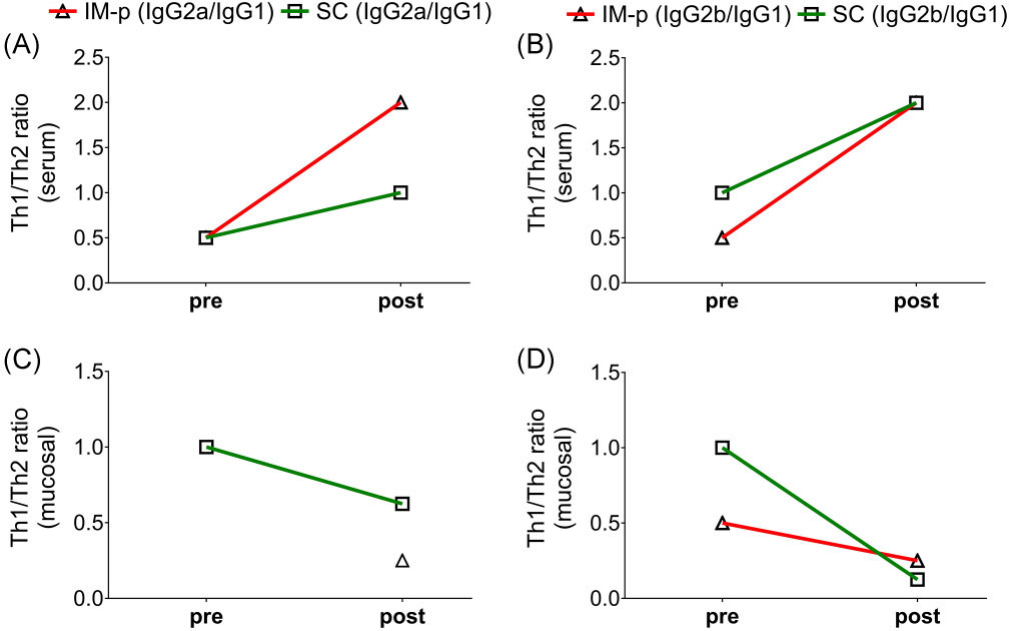
Serum and mucosal wash Th1/Th2 antibody ratios after immunization (pre) and challenge (post). Groups of mice were immunized and challenged, as described in Fig. 1 legend. Sera collected from groups of immunized (pre), and immunized-challenged (post) mice were pooled per group and used to quantify rMOMP-specific antibody isotypes by ELISA. Serum and mucosal antibodies endpoint titers were used for calculating the Th1/Th2 ratios; (A) and (B) serum, (C) and (D) mucosal wash.

Evaluation of the Th1/Th2 ratios after immunization in the SC and IM-p mice revealed that their IgG2a/IgG1 ratios (pre) were similar and suggestive of a Th2-type response, which skewed toward a Th1-type response after challenge (post) for the IM-p and not SC mice (Fig. 8A). The IgG2b/IgG1 ratios (pre) were similarly of the Th2-type in immunized (pre) mice, which skewed toward the Th1-type after challenge (post) in the SC and IM-p mice (Fig. 8B). On the contrary, mucosal Th1/Th2 antibody ratios after immunization (pre) were indicative of a Th1-type that were dominated after challenge (post) by Th2-type response (Fig. 8C and D). IgG2a production (pre) did not reach an endpoint titer to calculate the IgG2a/IgG1 ratio for the IM-p mice (Fig. 8C; Table 2). The rMOMP immunization induced lower Th1 responses and were not changed after a Cm challenge, except IgG2b (serum) and IgG2a (wash). PBS and rMOMP were not included in the graphs for clarity (Fig. 8). Overall, these results show that the SC and IM-p prime-boost immunizations induces Th1 response, whereas genital challenge because of bacteria skews it to a Th2 response (mucosal).

### Nanovaccine-induced antigen-specific avidity serum antibodies in mice

**Figure 9.**
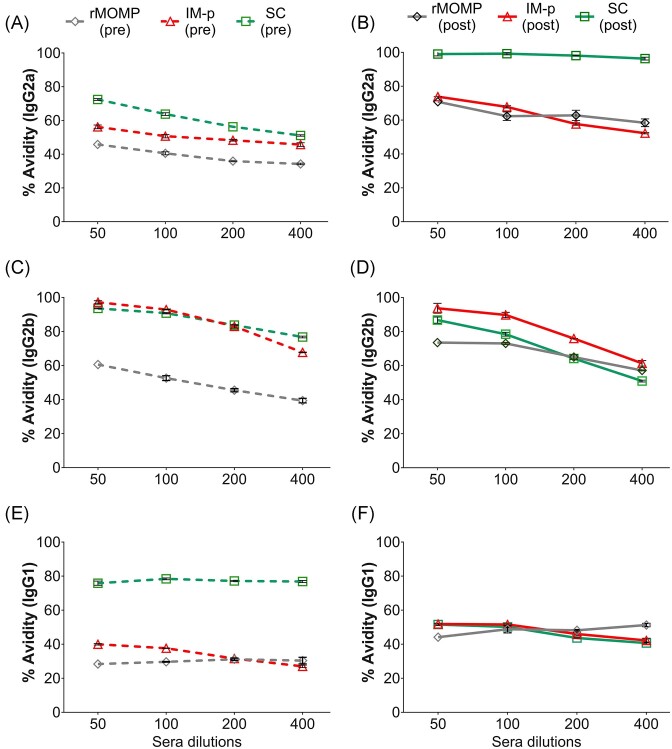
Antigen-specific serum IgG2a, IgG2b, and IgG1 avidity antibodies. Groups of mice were immunized and challenged, as described in the legend of Fig. 1. Avidity-ELISAs were conducted using pooled sera from immunized (pre) and immunized-challenged (post) mice to determine the avidity index (%) for rMOMP-specific (pre) (A) IgG2a, (C) IgG2b, and (E) IgG1 antibodies and post (B) IgG2a, (D) IgG2b, and (F) IgG1 antibodies. Each data point represents the mean ± SD of triplicate samples.

The avidity or functional affinity of antigen-specific antibodies induced by an effective vaccine renders specificity for the inactivation of the pathogen. The boosting of antigen-specific antibodies by PLGA-rMOMP in immunized mice, especially SC, led us to measure the avidity of serum IgG isotypes as a correlate of the humoral protective immunity. We used urea at a molar concentration of 8 M as a chaotropic agent to release the low-affinity antibodies from antigen-antibody complexes. Our results show an increase of IgG2a avidity after immunization (pre) being higher in SC and then IM-p, and rMOMP (Fig. 9A), with further increases after a genital challenge (post), notably in SC mice (Fig. 9B). Both SC and IM-p mice (pre) had similar and higher IgG2b avidity as compared to rMOMP (Fig. 9C), which slightly decreased in the SC, remained unchanged in IM-p, but increased in the rMOMP mice (post) after challenge (Fig. 9D). IgG1 avidity in the SC mice (pre) was high in comparison to the IM-p or rMOMP mice (Fig. 9E), which then drastically reduced in the SC, but slightly increased in the IM-p and rMOMP mice following challenge (post) (Fig. 9F). These results show that immunization with PLGA-rMOMP elicited high avidity antibodies. The high avidity Th1 antibodies produced by the SC homologous prime-boost may correlate as a measure of their biological functions in the humoral protective immunity of mice against a genital challenge.

### Nanovaccine-induced serum antibodies neutralization of EB

**Figure 10.**
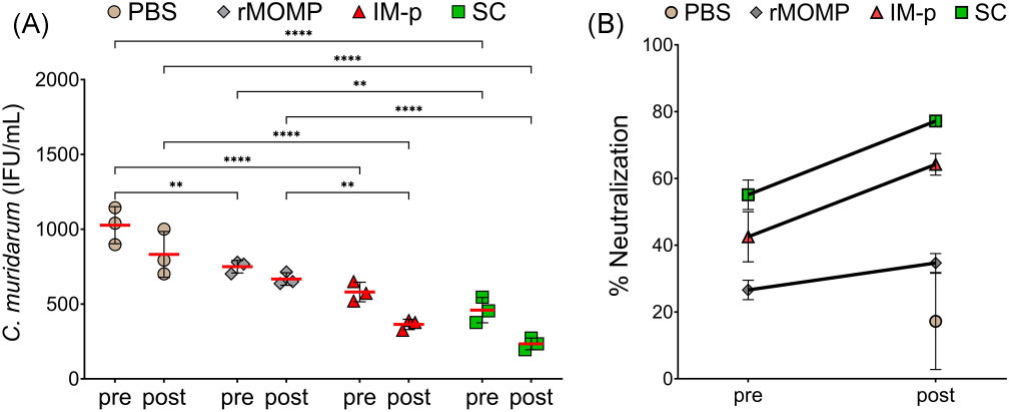
PLGA-rMOMP nanovaccine-induced serum neutralizing antibodies. Groups of mice were immunized and challenged as shown in the legend of Fig. 1. Pooled sera from immunized (pre) and immunized-challenged (post) mice were collected to determine their neutralization of EBs *in vitro*. McCoy cells were infected with sera-pretreated EBs and incubated for 30 h. Cells were fixed, stained, and observed under immunofluorescent microscope to count IFUs from three fields of one well. (A) Cm neutralization results are shown as IFU/ml. Each symbol represents mean ± SD of IFU counts from triplicate wells. The horizontal red line represents mean IFU/ml from each group. (B) The % neutralization (mean ± SEM) of Cm EB. Statistical analyses were performed using two-way ANOVA followed by Tukey’s multiple comparisons and significant differences were considered at **P < .01 and ***P < .0001.

Neutralizing antibodies bind to the surface of EBs and prevent the infectivity of the cells. Therefore, assessment of neutralizing antibodies from sera of immunized (pre) and immunized-infected (post) mice was performed *in vitro*. EBs were preincubated with sera and then added to the confluent layer of McCoy cells, following 30 h incubation to allow the development of inclusions. The results (Fig. 10A) show that the SC or IM-p immunization (pre) induced antibodies that significantly (*P* < .0001) neutralized Cm EB, when compared to the PBS group. Moreover, sera from both SC and IM-p mice (post) were significantly (*P* < .0001) more effective in neutralizing Cm EB in comparison to the non-immunized PBS (post) group. Even though, the sera from rMOMP-immunized (mice pre) significantly (*P* < .1) neutralized Cm EB, there was no significant increase after challenge (post). In addition, the rMOMP sera showed significantly (*P* < .01 and *P* < .0001) less neutralization of EB compared to those of the SC (pre and post) and (*P* < .1) IM-p groups (post).

We also analyzed the immunized (pre) and immunized-challenged (post) % sera neutralization of EB (Fig. 10B) in each group of mice. The analysis shows that SC and IM-p (pre) sera neutralized chlamydial EB by 55% and 42%, respectively, compared to the PBS group. Moreover, the SC and IM-p immunized-challenged (post) sera enhanced the neutralization of EB by 77% and 64%, respectively. rMOMP-immunized mice (pre) showed 26% neutralization with an increase to 34% after challenge (post) compared to the PBS group. Overall, the results show that the SC and IM-p immunization routes induce neutralizing antibodies. However, SC-route induced more than 20% additional neutralizing antibodies compared to the IM-p route.

## Discussion

Overwhelming research efforts to develop a subunit vaccine against Ct has encountered significant hurdles, including delivery platforms, adjuvants, and administration routes (Ralli-Jain et al. 2010, Fiorino et al. 2013, Dixit et al. 2014, Stary et al. 2015), to elicit efficacious protective immunity. Routes of vaccine administration are critical for inducing efficient cell-mediated and humoral immune responses to protect against a pathogen. Importantly, protection against Ct is dependent on robust CD4^+^ T-cells and antibody effector responses for bacterial clearance (Farris et al. 2010). An effective immunization strategy may require prime-boost delivery routes to influence the persistence of antigens and enhance T- and B-cell responses (Zacharias et al. 2018). Studies have shown that the SC route administration is considered appropriate for delivering antigens due to efficient draining to lymphoid organs to activate immune responses (Zhao et al. 2023). Notably, the SC route is gaining attention for the extended delivery of drugs and subunit vaccines using biodegradable nanoparticles (Taha et al. 2012, Van de Ven et al. 2012, Fairley et al. 2013, Zakeri-Milani et al. 2013, Dixit et al. 2014, Singh et al. 2015, Verma et al. 2018, Sahu et al. 2020). Likewise, subunit vaccines are also paving the way toward an IM immunization route that is more widespread for commercial vaccines (Herzog 2014, Ols et al. 2020) due to ease of administration and acceptability. Recently, Ols et al. (2020) demonstrated in nonhuman primates that the SC and IM immunization routes induced early differences in HIV-1 glycoprotein antigen trafficking with SC delivering to primary and IM to secondary lymph nodes (LNs); however, with similar induction of antigen-specific cellular and humoral responses.

A variety of immunization routes are being tested for a Ct vaccine (Badamchi-Zadeh et al. 2016, Wern et al. 2017), albeit some may undeniably and humanly impose challenges. Therefore, vaccines preferentially employing the most common immunization routes approach are always desirable. We previously reported that SC immunization of our chlamydial PLGA-rMOMP nanovaccine induced robust adaptive immune responses in mice along with memory and effector formation (Sahu et al. 2020), but did not afford complete protection of mice against a Cm genital challenge (Sahu et al. 2021). Given that vaccine delivery routes can appreciably impact the outcome of efficacious protective immune responses, here in the present study, we explored two prime-boost immunization strategies to compare the chlamydial nanovaccine-induced immunogenicity and protective efficacy in mice against a chlamydial genital challenge.

We have demonstrated here that SC (homologous) and IM-p (heterologous) prime-boost immunization strategies effectively protected mice against genital *Chlamydia* by enhancing an early bacterial clearance and reduced bacterial burdens in contrast to the rMOMP and control mice. However, the IM-p mice had higher bacterial burdens, probably due to a lack of tolerance against establishing a Cm infection. Conversely, the SC mice prevented the establishment of infection by the reduced bacterial burden and early clearance of infection with total of 88% IFU reduction, suggesting enhanced protection afforded by the homologous immunization. Ralli-Jain et al. (2010) investigated systemic (IM and SC) and mucosal (sublingual and colonic) immunization routes, alone and in combinations using chlamydial rMOMP, against a respiratory chlamydial challenge. Their results revealed that the combined mucosal and systemic routes were most effective, especially a simultaneous combination of the sublingual, IM, and SC routes (Ralli-Jain et al. 2010). Similarly, Carmichael et al. (2011) reported that a combined systemic and intravaginal mucosal route enhanced protection against a *Chlamydia* genital challenge. However, the mucosal routes require a high antigen dose for immunization due to rapid clearance (de la Maza et al. 2017). Ideally, mucosal routes are more favorable for mucosal pathogens to induce local protective immune effectors. Indeed some preclinical vaccines against mucosal pathogens do employ mucosal routes such as intranasal for *Yersinia pestis* (Wang et al. 2020), influenza virus (Quan Le et al. 2020), and *Leishmania* (infantum) *chagasi* (Leal et al. 2015). Oral immunization is also acceptable for some vaccines, i.e. the poliovirus vaccine (Holmgren and Czerkinsky 2005). Several *Chlamydia* vaccines in preclinical development similarly are being administered via the mucosal route (Pal et al. 1996, Manam et al. 2013). Herein, our findings reveal a paradigm shift from a mucosal pathogen mandating a mucosal route for vaccine administration and efficacy, which could be attributed to the PLGA delivery system’s versatility for various immunization routes to elicit protection against genital *Chlamydia*. Earlier research indicated mandatory requirements for T-cells with a minimal role for antibodies in protective immunity and clearance of *Chlamydia* (Su and Caldwell 1995, Li et al. 2008). Now compelling evidence reveals that protection against *Chlamydia* requires cell-mediated and humoral immune effectors for bacterial clearance (Farris et al. 2010).

The role of antigen-specific CD4^+^ T-cells producing Th1 cytokines, particularly IFN-γ is well-recognized in the clearance of *Chlamydia* (Lin et al. 2019, Tifrea et al. 2020). In the current study, we observed that T-cells from SC and IM-p mice secreted higher levels of rMOMP-specific IFN-γ after immunization and challenge compared to the rMOMP and control mice. IFN-γ production accompanied by IL-2 is a clear indicator of T-cell proliferation and is congruent with our previous reports of PLGA-rMOMP (Sahu et al. 2021) or PLA-PEG-M278 (a peptide of MOMP) (Verma et al. 2018) immunization of mice via the SC-route that exhibited a predominant Th1 immune response. The presence of IL-2 along with IFN-γ is a dynamic relationship as IL-2 directly acts to stimulate T-cells to produce IFN-γ (Kasahara et al. 1983), a requisite cytokine for protection against *Chlamydia* (Helble et al. 2020). Therefore, the enhanced IL-2 and IFN-γ production in the current study underscores the described relationship in the above study. We also observed an upregulated production of the immunoregulatory IL-17 cytokine in the SC, IM-p, and rMOMP immunized mice (Pre) facilitated by the PLGA-rMOMP nanovaccine, and in all groups of mice after Cm genital challenge, which is similar to our previous findings (Sahu et al. 2021). IL-17 is a common cytokine produced by Th17 T-cells, however its role in *Chlamydia* infection is still debatable between protection and pathology. Andrew et al. (2013) study demonstrated that IL-17 KO mice that were infected with *Chlamydia* and then immunized intranasally with MOMP, cholera toxin and CpG adjuvant were unable to clear the infection and exhibited less pathology, IFN-γ production, and T-cell proliferation. Upregulation of IL-17 producing T-cells also has been linked with IL-6 producing T-cells as reported by Moore-Connors et al. (2013) and Zhou et al. (2013) in *Chlamydia*. The protection afforded by SC and IM-p groups of mice may be correlated with IL-6 regulation of IL-17 coupled with increased T-cell producing IFN-γ, which are induced by IL-2 proliferation. It can be said that IL-17 plays an important role in protection but may not be directly associated with pathology.

We also observed that immunized and protected mice produced IL-4, IL-9, and IL-13, all Th2 cytokines, albeit at lower levels compared to the predominant levels of the Th1 cytokines, IFN-γ and IL-2, which underscores our previous observation for immunization via SC or IN routes (Sahu et al. 2021). In addition, elevated levels of G-CSF were seen in SC mice (pre) followed by IM-p, which were further increased after Cm challenge, as well as in other groups of mice. In general, G-CSF functions as survival, proliferation and differentiation of neutrophils and inducing leukocytes cell production from bone-marrow (Link 2022) but its role in *Chlamydia* is yet to be defined. Of note, SC and IM-p mice exhibited rMOMP-specific CD4^+^ T-cell activation, proliferation, and differentiation into memory (CD44^high^ CD62L^high^) phenotypes but with a higher effector (CD44^high^ CD62L^low^) phenotype in SC, which is consistent with our previous report (Verma et al. 2018, Sahu et al. 2021) and others (Li et al. 2008, Helble et al. 2020). Interestingly, CCL-2 production (Fig. 3C) in SC or IM-p mice (pre) is additional evidence of inducing recruitment of memory T-cells, especially IFN-γ producing T-cells. It was reported that *Chlamydia* induce CCL-2 production (Belay et al. 2002, Schrader et al. 2007), however, CCL-2 production by SC and IM-p immunization is an interesting finding in the current study. The differences in cellular responses between SC and IM-p mice may infer differences in antigen processing, as demonstrated in nonhuman primates with SC targeting primary LNs and IM secondary LNs (Ols et al. 2020). Our recent publication revealed that PLGA-rMOMP increased MHC class II antigen presentation and targeted primary LNs in SC-immunized mice (Sahu et al. 2020). Collectively, we could speculate that efficient processing with higher cell-mediated immune effectors could, in part, explain the better protection of SC than the IM-p mice against genital *Chlamydia*.

That antibodies are essential for the clearance of *Chlamydia* was elegantly demonstrated by Farris et al. (2010) in B-cell deficient mice lacking vaccine-induced protection and by Pal et al. (2005) linking *Chlamydia*-specific Th1 (IgG2a and IgG2b) antibodies to protection. Herein, protection of the SC and IM-p mice involved the engagement of Th1 (IgG2a and IgG2b) and Th2 (IgG1) antibodies, given their high antibody titers, especially in SC mice. Interestingly, Th2-type antibody responses dominated after immunization with a bias toward Th1-type after challenge, facilitated by the bacterial infection. After immunization, the dominant Th2 antibodies may have ensued from the self-adjuvanticity of PLGA since mice immunized with rMOMP induced mainly Th1 antibodies. Even though, we observed a reduction in rMOMP-specific systemic antibodies titer after Cm challenge in SC mice, this contrasted with the IM-p mice. Nevertheless, SC and IM-p mice induced mixed Th1/Th2 antibodies, but only Th1 antibodies exhibited high avidity, especially in SC mice. This finding, further correlates with Th1 isotypes preventing establishment of early infection (Hawkins et al. 2002, Ralli-Jain et al. 2010) and supposedly the differences in the protection levels between the IM-p and SC mice in clearing genital *Chlamydia*.

In this study, higher rMOMP-specific IgA occurred in the IM-p than SC mice, a pattern we previously observed in PLGA-rMOMP SC-immunized mice (Sahu et al. 2021). Results from studies indicate antigen-specific IgA can only provide a partial reduction of chlamydial infections (Armitage et al. 2014, Erneholm et al. 2019). Armitage et al. (2014) revealed that rMOMP-specific IgA antibodies reduced chlamydial infection by 44% in the absence of CD4^+^ T-cells. There is documentation of IgA and IgG producing plasma cells in the genital tract of pigs following an IM vaccination with UV-inactivated bacteria/CAF01 and a *Chlamydia* intravaginal challenge (Erneholm et al. 2019). A vaccine developed by Jiang et al. (2017) comprised of multiepitopes peptides of MOMP with Hepatitis B virus core antigen (HBcAg) enhanced immunogenicity with increased IFN-γ, IgG, and IgA effectors that improved efficacy and clearance of genital infection earlier than the controls. Presumably, the protective immunity against *Chlamydia* infection requires a synergistic effort facilitated by cellular and humoral immune responses, as shown here, since specific antibodies only partially reduce the *Chlamydia* mucosal burden (Darville et al. 2019).

Onset of neutralizing antibodies to clear the pathogen directly correlates with memory B-cells (Young and Brink 2021). As a result, these memory cells are responsible for inducing humoral protective responses against pathogen and are important for an efficacious vaccine. Serum antibody-mediated *Chlamydia* neutralization *in vitro* is an important tool to predict the effectiveness of a *Chlamydia* vaccine (de la Maza et al. 2017). There are multiple reports indicating that promising *Chlamydia* vaccine candidates induce neutralizing antibodies (Olsen et al. 2021, Tifrea et al. 2021, Zuo et al. 2021). Our results show that the levels of EB neutralization enhanced after the Cm intravaginal challenge, which are similar to our previous studies (Verma et al. 2018, Sahu et al. 2021). Chlamydial MOMP is the most proposed vaccine candidate for *Chlamydia* (Sahu et al. 2021, Huynh et al. 2022, Pal et al. 2023) and have shown that MOMP-specific antibodies possess neutralization functionality (Collar et al. 2022). In this study, we demonstrated that the nanovaccine homologous (SC) route is superior over the heterologous (IM-p) priming immunization route against a Cm genital challenge.

## Conclusions

In conclusion, the homologous SC prime-boost immunization of mice with PLGA-rMOMP induced higher cell-mediated and humoral immune responses and conferred better protection against a Cm genital challenge compared to the heterologous IM-p. This study is the first to report the comparison of IM-p versus SC prime-boost immunization routes for immunogenicity and protective efficacy in *Chlamydia* vaccine development strategy. With further optimization, perhaps including an adjuvant, PLGA-rMOMP holds promise as a nanovaccine candidate that can confer even higher protection against genital chlamydial infections.

## Supplementary Material

ftae004_Supplemental_File

## Data Availability

The original contributions presented in the study are included in the article. Further inquiries can be directed to the corresponding author.
