# Supplementary material for: Effects of prime-boost strategies on the protective efficacy and immunogenicity of a PLGA (85:15)-encapsulated Chlamydia recombinant MOMP nanovaccine
Source: Pathog Dis. 2024 Jun 11;82:ftae004. doi: 10.1093/femspd/ftae004 (PMC11186516; doi:10.1093/femspd/ftae004)
Supplement: ftae004_Supplemental_File [file ftae004_supplemental_file.pdf]

Supplementary Figure 1.

Unstained (Naïve)

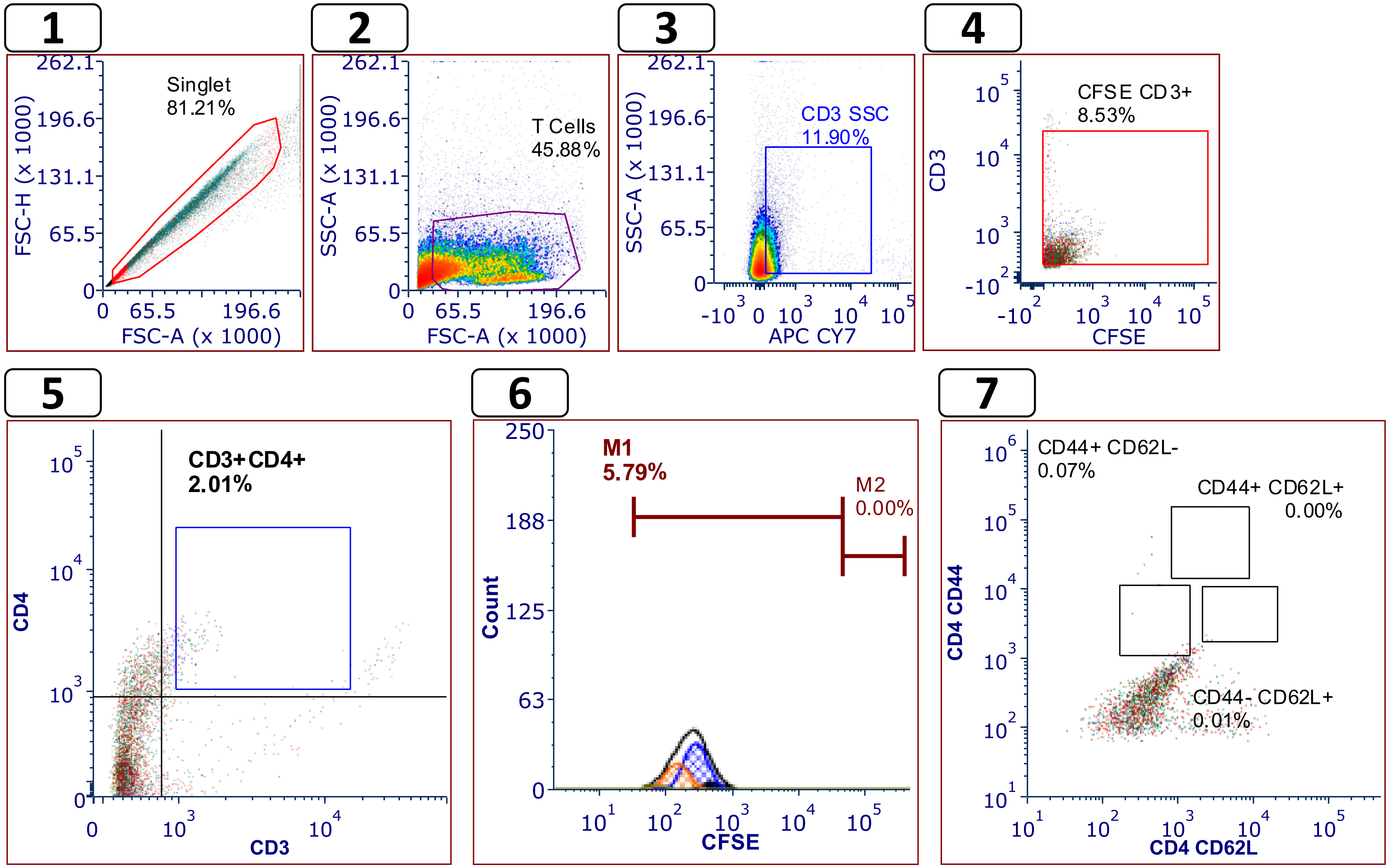

The gating sequence is indicated as numbers (1 through 7)

The gating strategies correlate with Figure 5 (A through L) pre samples

Supplementary Figure 2.

CFSE stained (Naïve)

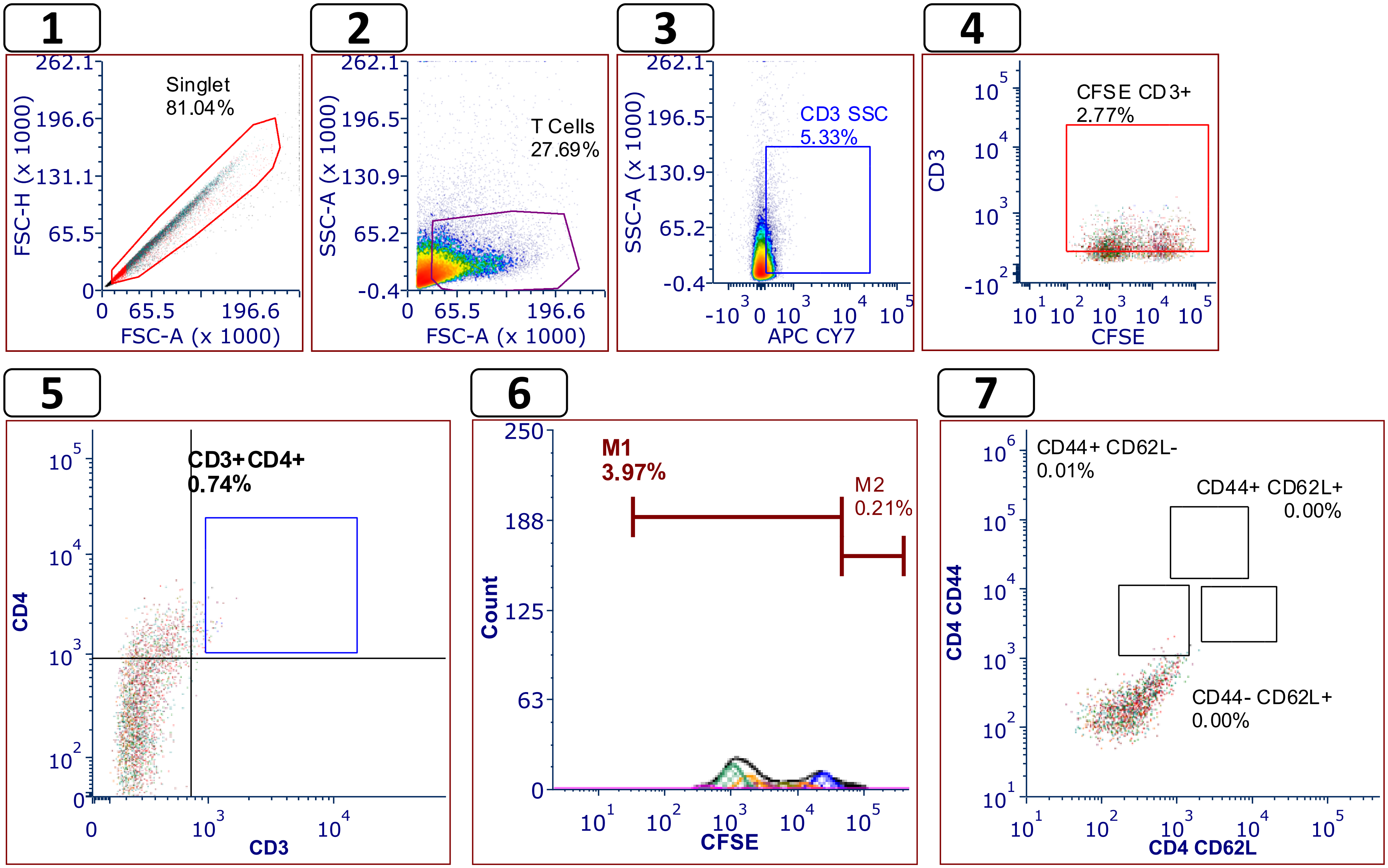

The gating sequence is indicated as numbers (1 through 7)

The gating strategies correlate with Figure 5 (A through L) pre samples

Supplementary Figure 3.

Con A stimulated (Naïve)

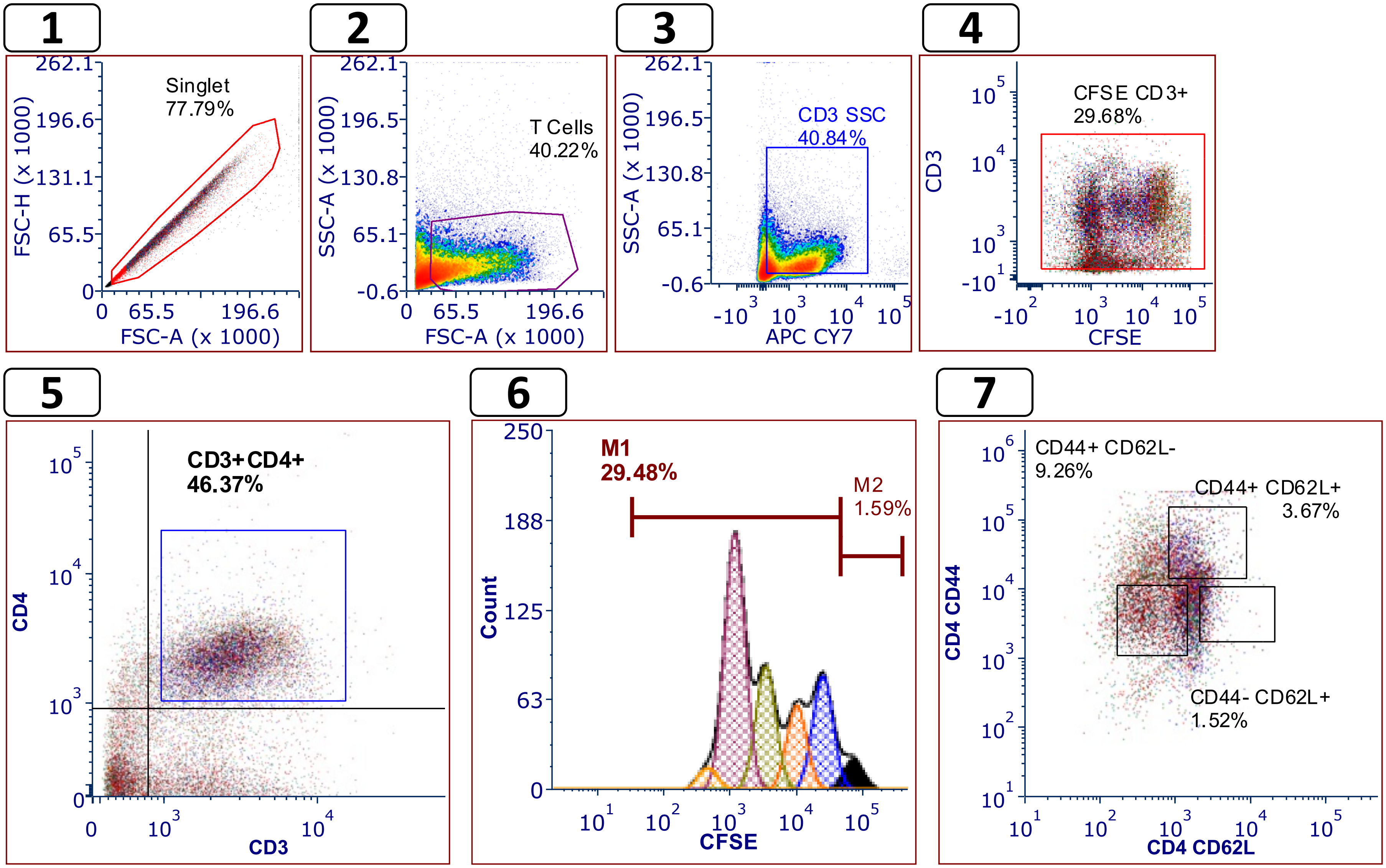

The gating sequence is indicated as numbers (1 through 7)

The gating strategies correlate with Figure 5 (A through L) pre samples

Supplementary Figure 4.

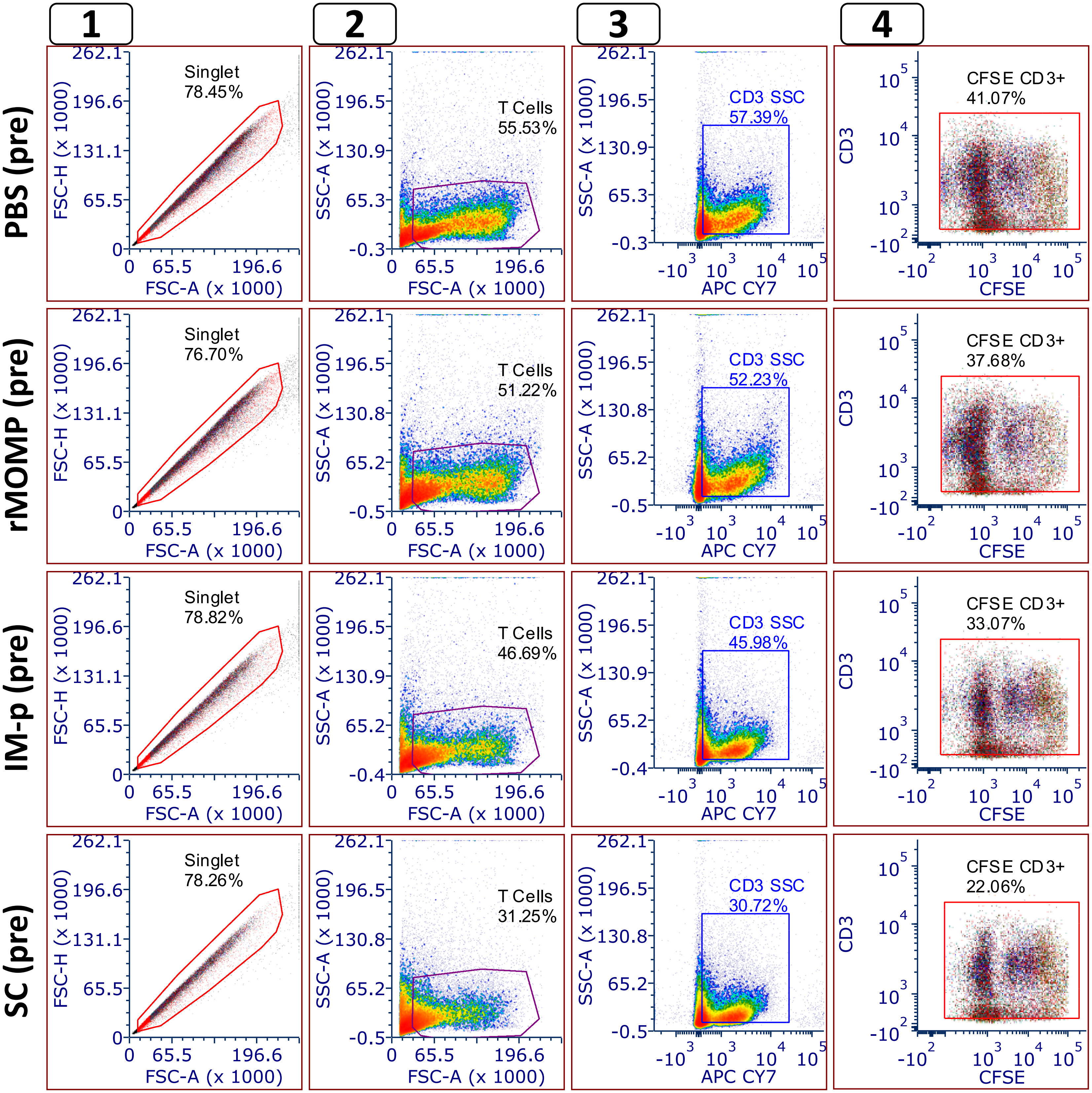

The gating sequence is indicated as numbers (1 through 4) and 5 through 7 in the main manuscript (Figure 5A through L) pre samples

Supplementary Figure 5.

Unstained (Naïve)

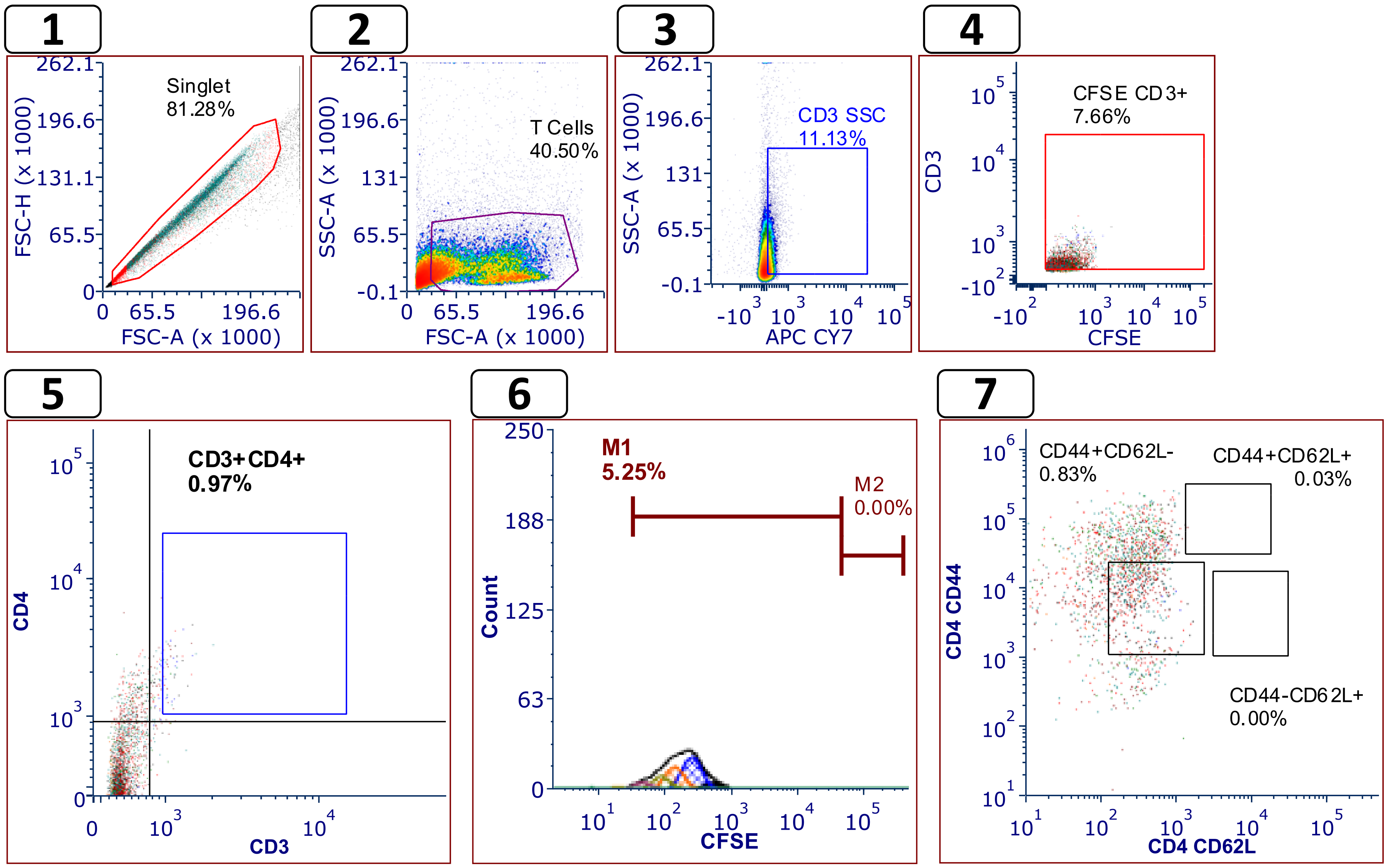

The gating sequence is indicated as numbers (1 through 7)

The gating strategies correlate with Figure 5 (M through X) post samples

Supplementary Figure 6.

CFSE stained (Naïve)

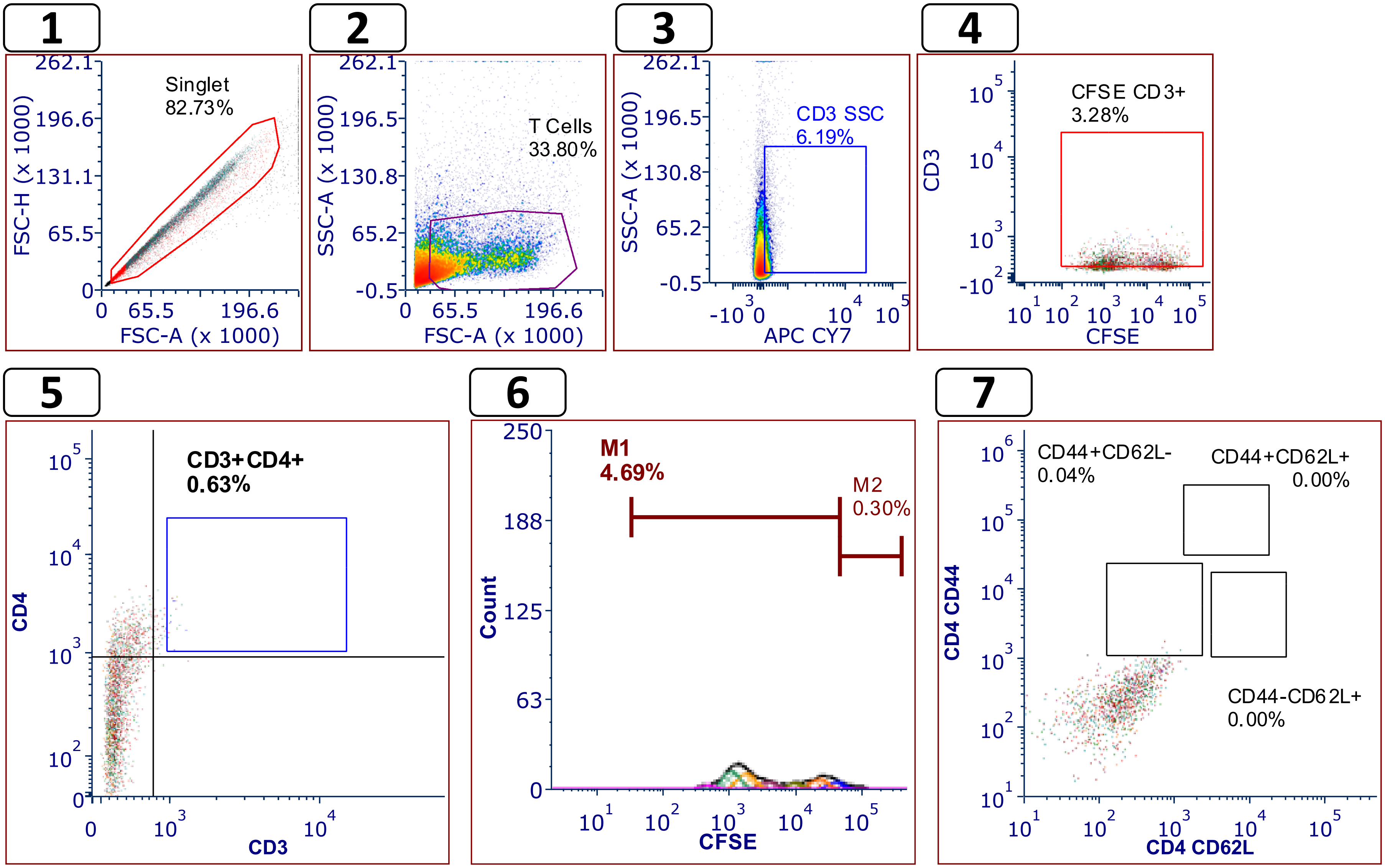

The gating sequence is indicated as numbers (1 through 7)

The gating strategies correlate with Figure 5 (M through X) post samples

Supplementary Figure 7.

Con A stimulated (Naïve)

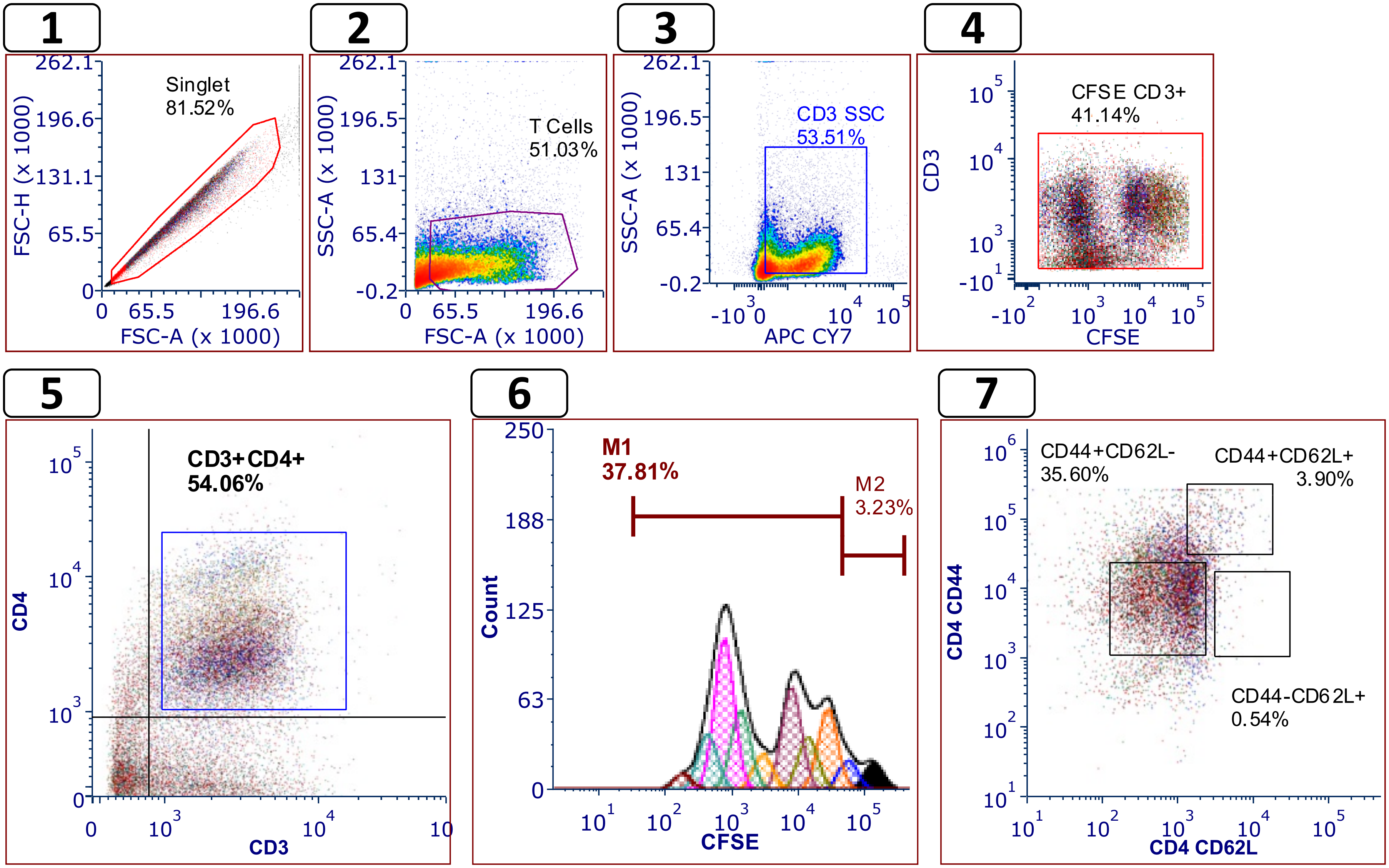

The gating sequence is indicated as numbers (1 through 7)

The gating strategies correlate with Figure 5 (M through X) post samples

Supplementary Figure 8.

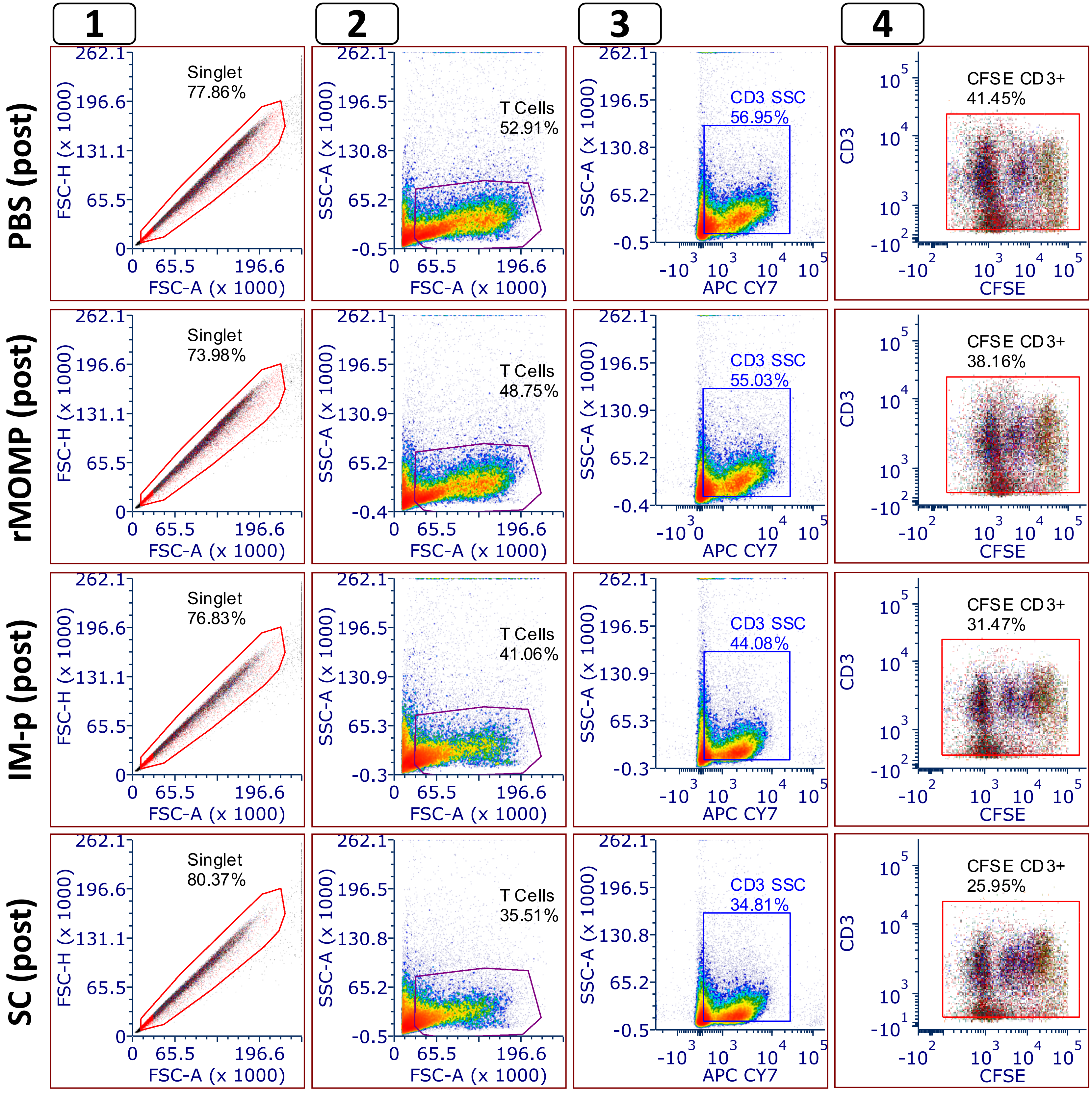

The gating sequence is indicated as numbers (1 through 4) and 5 through 7 in the main manuscript (Figure 5M through X)
